# A Lead-Based Fragment Library Screening of the Glycosyltransferase WaaG from *Escherichia coli*

**DOI:** 10.3390/ph15020209

**Published:** 2022-02-09

**Authors:** Federico Riu, Alessandro Ruda, Olof Engström, Claudio Muheim, Hani Mobarak, Jonas Ståhle, Paul Kosma, Antonio Carta, Daniel O. Daley, Göran Widmalm

**Affiliations:** 1 Department of Medical, Surgical and Experimental Sciences, University of Sassari, Via Muroni, 23A, 07100 Sassari, Italy; federicoriu1@gmail.com (F.R.); acarta@uniss.it (A.C.); 2 Arrhenius Laboratory, Department of Organic Chemistry, Stockholm University, S-106 91 Stockholm, Sweden; alessandro.ruda@su.se (A.R.); j.olof.engstrom@gmail.com (O.E.); hani@gmx.se (H.M.); jstaahle@gmail.com (J.S.); 3 Arrhenius Laboratory, Department of Biochemistry and Biophysics, Stockholm University, S-106 91 Stockholm, Sweden; clamuheim@gmail.com (C.M.); daniel.daley@dbb.su.se (D.O.D.); 4 Department of Chemistry, University of Natural Resources and Life Sciences—Vienna, 1190 Vienna, Austria; paul.kosma@boku.ac.at

**Keywords:** molecular docking, molecular dynamics, binding free energy, NMR spectroscopy

## Abstract

Glucosyl transferase I (WaaG) in *E. coli* catalyzes the transfer of an α-d-glucosyl group to the inner core of the lipopolysaccharide (LPS) and plays an important role in the biogenesis of the outer membrane. If its activity could be inhibited, the integrity of the outer membrane would be compromised and the bacterium would be susceptible to antibiotics that are normally prevented from entering the cell. Herein, three libraries of molecules (A, B and C) were docked in the binding pocket of WaaG, utilizing the docking binding affinity as a filter to select fragment-based compounds for further investigations. From the results of the docking procedure, a selection of compounds was investigated by molecular dynamics (MD) simulations to obtain binding free energy (BFE) and *K*_D_ values for ligands as an evaluation for the binding to WaaG. Derivatives of 1,3-thiazoles (**A7** and **A4**) from library A and 1,3,4-thiadiazole (**B33**) from library B displayed a promising profile of BFE, with *K*_D_ < mM, viz., 0.11, 0.62 and 0.04 mM, respectively. Further root-mean-square-deviation (RMSD), electrostatic/van der Waals contribution to the binding and H-bond interactions displayed a favorable profile for ligands **A4** and **B33**. Mannose and/or heptose-containing disaccharides **C1–C4**, representing sub-structures of the inner core of the LPS, were also investigated by MD simulations, and compound **C4^2−^** showed a calculated *K*_D_ = 0.4 µM. In the presence of UDP-Glc^2−^, the best-docked pose of disaccharide **C4^2−^** is proximate to the glucose-binding site of WaaG. A study of the variation in angle and distance was performed on the different portions of WaaG (N-, the C- domains and the hinge region). The Spearman correlation coefficient between the two variables was close to unity, where both variables increase in the same way, suggesting a conformational rearrangement of the protein during the MD simulation, revealing molecular motions of the enzyme that may be part of the catalytic cycle. Selected compounds were also analyzed by Saturation Transfer Difference (STD) NMR experiments. STD effects were notable for the 1,3-thiazole derivatives **A4**, **A8** and **A15** with the apo form of the protein as well as in the presence of UDP for **A4**.

## 1. Introduction

Pathogenic multi-drug resistant (MDR) strains of gram-negative bacteria are currently a major public health concern. They represent the causative agents of diseases such as gastroenteritis, infections of the urinary tract, the blood and the central nervous system [1]. The World Health Organization (WHO) has published a list of priority pathogens, highlighting the concern for the lack of treatment solutions for gram-negative pathogens, including *Escherichia coli* [2]. Furthermore, antibiotic resistance has been repeatedly reported as an alarming global issue [3], since bacteria easily spread across countries through goods, animals and people. It also represents a significant problem for major surgery and cancer treatment, which leads to large public health expenses.

Moreover, certain strains of *E. coli* have easily and quickly spread in the community. In 2011, some countries in the WHO European Region experienced a significant *E. coli* outbreak: the increasing cases of bloody diarrhea and hemolytic syndrome (HUS) were traced to infections from verocytotoxin-producing *E. coli* O104:H4 in Germany and in 15 other countries in Europe and North America [4]. Another consequence of *E. coli* resistance is the treatment of urinary tract infections with fluoroquinolone antibiotics. In many countries, this treatment is becoming increasingly ineffective [5]. Thus, there is a pressing need to find better ways to use the currently available antibiotics [6,7,8,9,10].

Bacteria develop antibiotic resistance in many different ways. It can be acquired through mutation and horizontal gene transfer, or it can be intrinsic (e.g., the strain is naturally insensitive to the antibiotic). The outer membrane in gram-negative bacteria is a major contributing factor to intrinsic resistance [11,12,13,14]. It acts as a selective permeability barrier that enables the entry of essential nutrients and ions whilst simultaneously preventing the entry of many classes of antibiotics into the cell [15,16].

**Figure 1:**
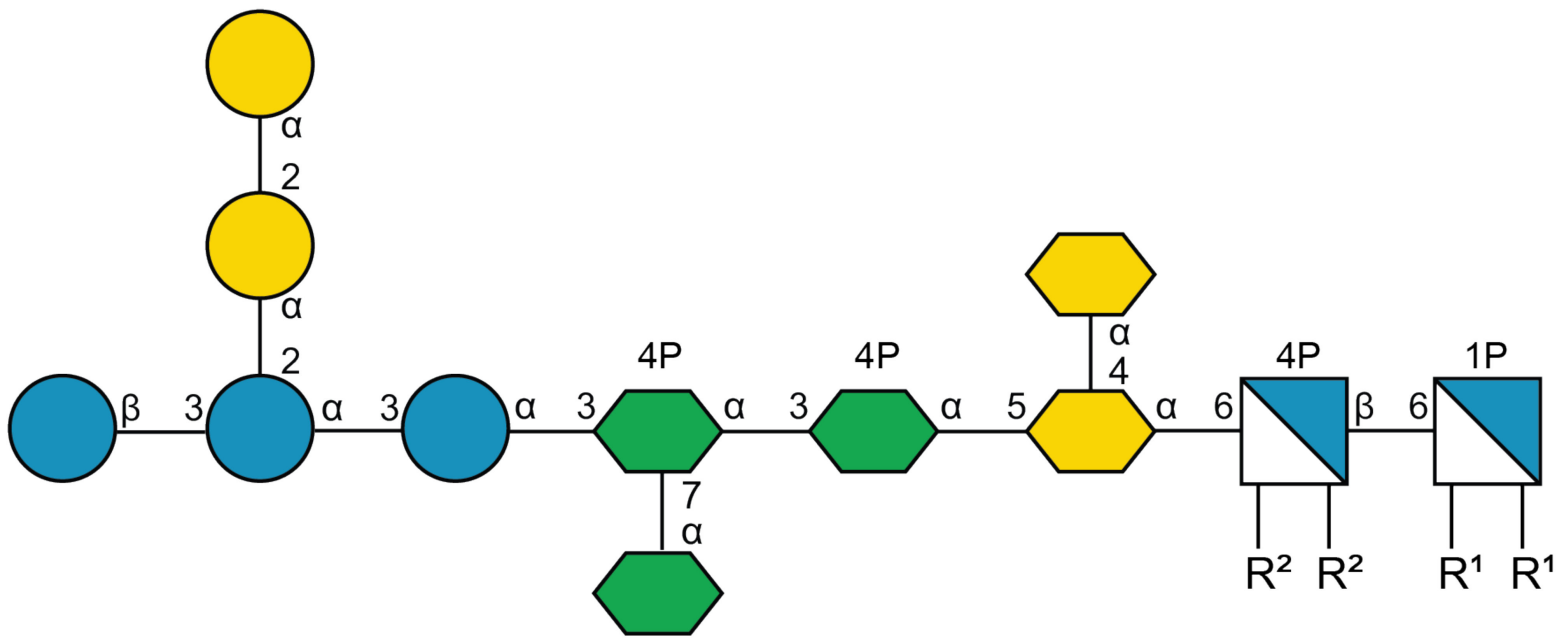
Structural representation of outer (to the left) and inner (middle section) portions of the R1 core and the lipid A (to the right) of LPS [20]. R^1^ and R^2^ represent acyl groups attached to glucosamine residues of Lipid A. The structure is reported utilizing the Symbol Nomenclature For Glycans (SFNG) representation [21].

As a first step to overcome intrinsic resistance in *E. coli*, we have investigated the possibility to inhibit glucosyl transferase I (WaaG or RfaG), a protein that catalyzes the transfer of an α-d-glucosyl group from UDP-Glc onto l-*glycero*-d-*manno*-heptose-II during the synthesis of the core region of the lipopolysaccharide (LPS) [17]. The general structure of LPS has come from different studies on various gram-negative bacteria. From a structural point of view, lipid A is the membrane-embedded lipophilic portion of LPS consisting of glucosamine dimers (d-GlcN), which are bound to acyl chains. Lipid A is attached to the core region containing Kdo (3-deoxy-d-*manno*-2-octulosonic acid) and l-*glycero*-d-*manno*-heptose (l,d-Hep), as well as some hexosamines and hexoses (Figure 1). The O-polysaccharides (or O-antigens) are, when present, linked to the core region. Significantly, deletion of the gene encoding WaaG destabilizes the outer membrane of *E. coli* by interfering with LPS core phosphorylation and results in truncated LPS immediately after the inner core heptose residues [18]. This perturbation changes the chemical structure of the LPS and makes *E. coli* more susceptible to different classes of antibiotics—for example, rifamycins and aminoglycosides [19].

**Figure 2:**
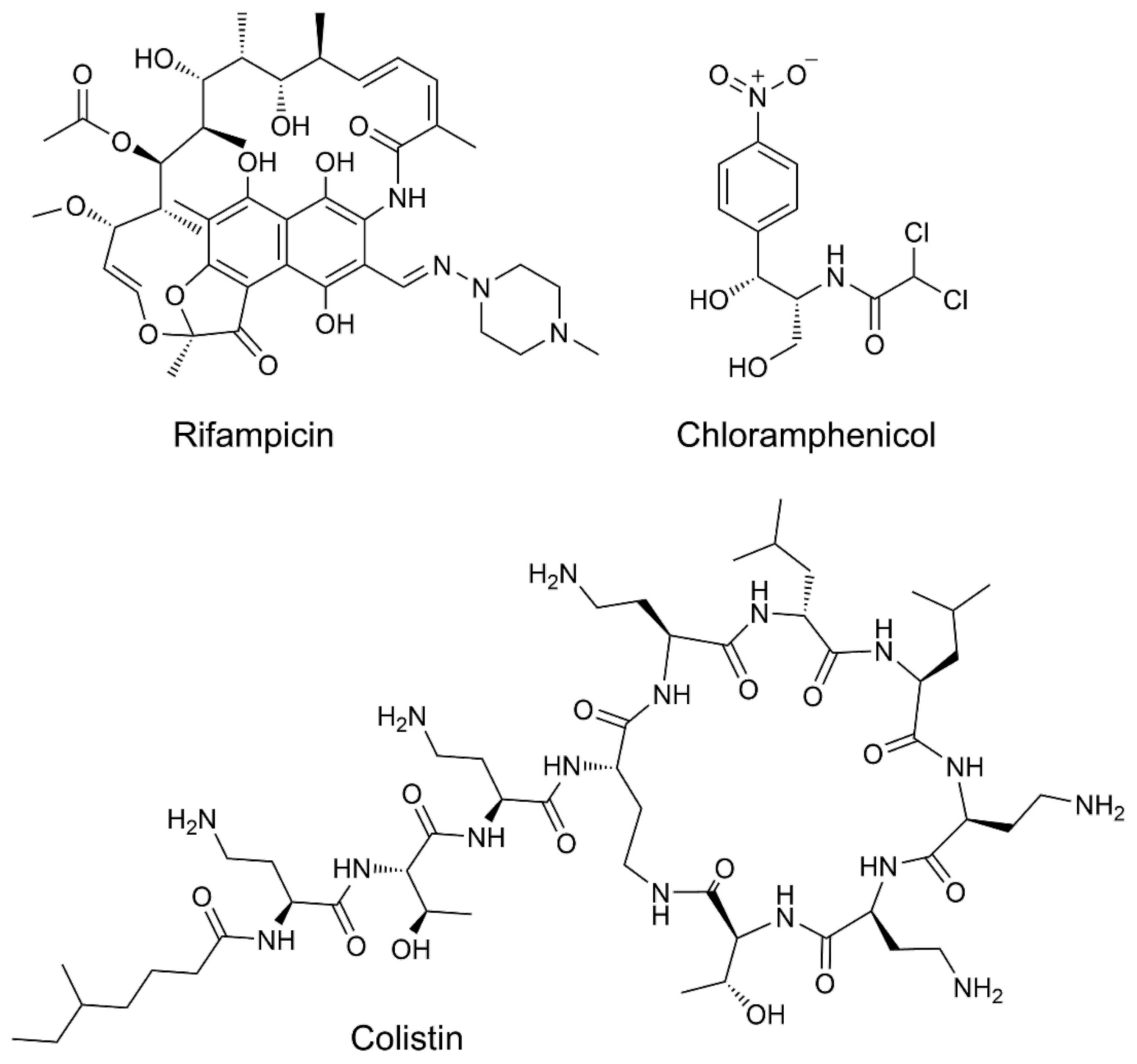
Structural formulas for rifampicin, chloramphenicol and colistin.

Small molecules that bind to WaaG in vitro could represent a strategic way to make *E. coli* more sensitive to different drugs, such as rifampicin, chloramphenicol and colistin (Figure 2). Colistin-resistant bacteria are becoming significantly untreatable because of their wide spreading [5]. The small organic molecules will serve as scaffolds for the design of more effective inhibitors of WaaG and could be used as co-drugs that potentiate the action of antibiotics in gram-negative bacteria.

Previous molecular docking and NMR studies elucidated the binding of natural ligands to WaaG, showing 0.6 mM and 9 µM as *K*_d_ values for uridine and UDP, respectively [22]. Three compounds (in this work reported as **A1**–**A3**) from an Ro3 Maybridge 2006 Library compliant with the ‘Rule of Three’ for fragment-based lead discovery, were selected through NMR spectroscopy screening and utilized for preliminary docking studies [22]. Compound **A1** inhibited WaaG with an IC_50_ = 1.0 mM, which opened the door for new structural modifications that act as potent inhibitors [23].

**Scheme 1:**
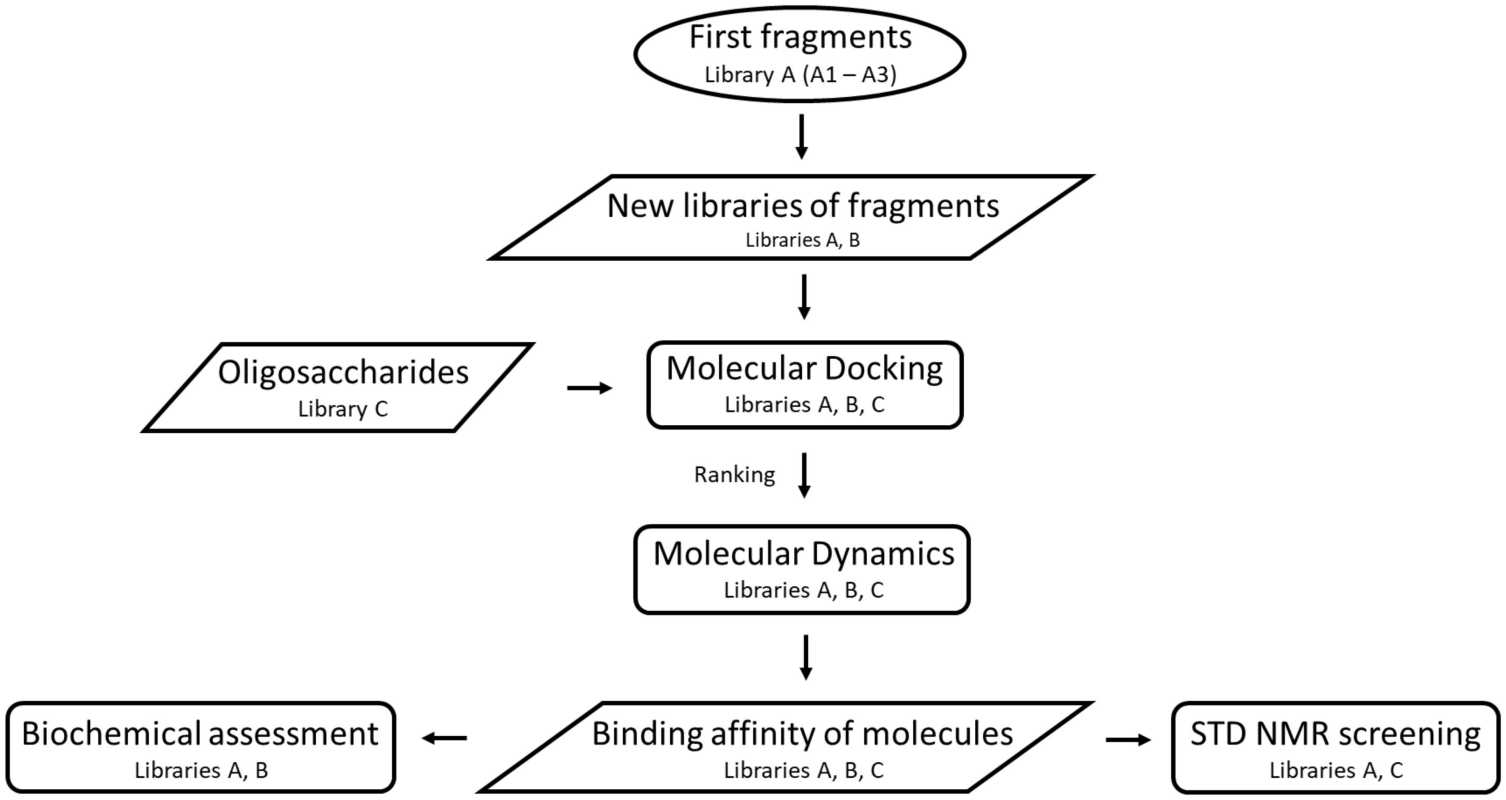
Flowchart of the computational and experimental study on WaaG and its interactions with compounds from libraries A, B and C.

In order to investigate leads to new inhibitors of WaaG, an initial fragment-based drug discovery (FBDD) process included the selection of different fragments (library A), small molecules (library B) and, for comparison, oligosaccharides (library C) (Scheme 1). Molecular docking and further molecular dynamics (MD) simulations were conducted on the best-docked poses of the most promising molecules. At a later stage, STD NMR (Saturation Transfer Difference Nuclear Magnetic Resonance) [24] spectroscopy and complementary experiments addressing inhibitory activity were carried out through in vitro biochemical assessment using libraries A and B. The STD NMR experiment is a robust and valuable method [25] for the determination of the binding of ligands to proteins [26] due to its versatility and low susceptibility to false positives. It requires only a small amount of unlabeled protein, and it is used in combination with docking studies to predict the conformational state of a ligand in a binding pocket [27].

## 2. Results

### 2.1. Construction of Libraries

The present study considers glycosyltransferase WaaG as the target for further fragment-based studies [28]. In the RCSB protein database (PDB), there is a deposited crystal structure of WaaG bound to UDP-2-fluoro-2-deoxy-d-glucose (U2F) with the PDB code 2iw1. This protein structure was utilized for further docking and molecular dynamics investigations, as well as to generate a model of UDP-Glc^2−^ bound to the protein.

In our previous study, the drug discovery fragment-based approach involved approximately 500 compounds from the Ro3 Maybridge 2006 library and STD NMR spectroscopy. Molecular docking and MD simulations were employed to evaluate their binding affinity for the active site of WaaG [22]. Three bicyclic compounds (**A1–A3**) were selected according to their binding activity towards WaaG, competing with UDP-Glc. The docking studies showed that uridine, UDP, **A1** and **A3** bind to the same pocket. In contrast, **A2** binds to the glucosyl region in the active site [22].

**Figure 3:**
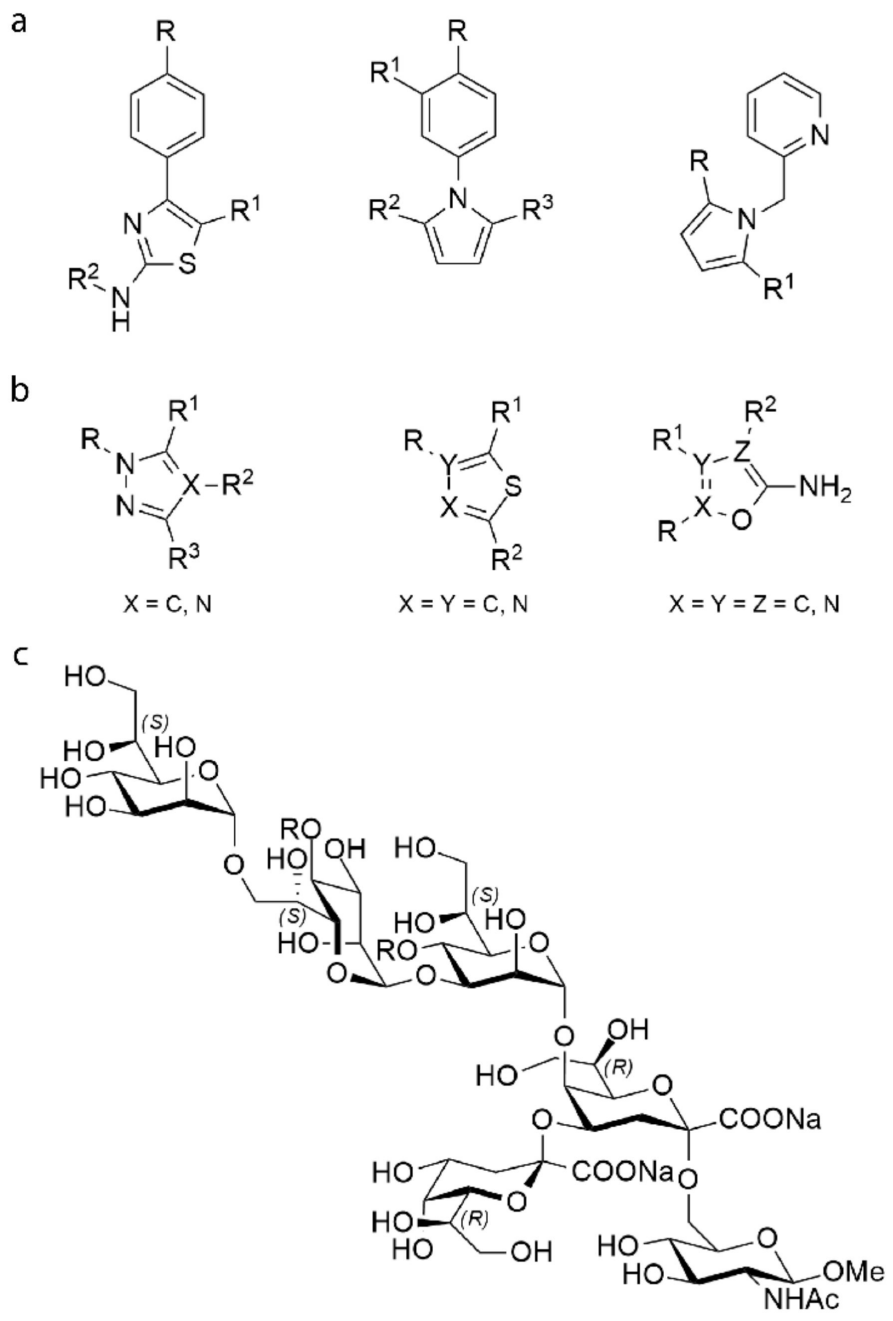
Chemical backbone structure of the ligands from library A (**a**) and B (**b**); in the carbohydrate-based library C (**c**) R = HPO_3_Na. Figures S1–S5 report all ligands of libraries A, B and C.

In this work, we present a fragment-based approach to find a new set of compounds that may inhibit the enzymatic activity of WaaG. The above-mentioned ligands **A1–A3** led to a first ligand library consisting of 20 hetero-bicyclic compounds (**A1–A20)** with a range of molecular weights between 158 and 257 Da (library A). The scaffolds of compounds **A1–A3** (phenylthiazole, phenylpyrrole and methylpyridine-pyrrole, respectively), were modified by adding or removing polar, e.g., hydroxyl, carboxyl, amide, aldehyde or nonpolar (e.g., methyl, ethyl, phenyl, halogen atoms) functional groups. Figure 3A shows a schematic representation of the scaffolds of the library, and Figure S1 depicts the chemical structure of each ligand. Further modifications involved the phenylthiazole scaffold transformed into an indole thiazole (**A14**). Moreover, the acetate salt of a phenylthiazole derivative was included in the library (**A13**).

In order to find drug-like inhibitors, a second chemical library, library B, was generated. Library B includes 37 larger fragments (**B1–B33**) structurally expanded from library A. They are different in terms of molecular weight (M_w_ = 176–391 Da). Some of these fragments are hetero-bicyclic molecules bearing a variety of heterocycle rings, e.g., triazole, thiadiazole. Figure 3B presents the different heterocycles included in the structure of the ligands. In order to enhance ligand-receptor affinity, ligands with higher M_w_ were devised through a fragment growing process [29]. Figures S2 and S3 show the structure of each ligand.

In order to study the interactions between WaaG, UDP-Glc (donor) and the acceptor site of the inner core of LPS, a set of oligosaccharides were included in a third library, library C (**C1–C12**). The structures of this library mimic the outer core of LPS to different extents. A schematic representation of the oligosaccharide structures is depicted in Figure 3C. The first acceptor compound is represented by α-d-Man*p*-(1→3)-α-d-Man*p*-OMe (**C1**). Four disaccharide compounds were designed to mimic building blocks of the LPS inner core: l-α-d-Hep*p*-(1→3)-l-α-d-Hep*p*-OMe (**C2**), l-α-d-Hep*p*-(1→3)-l-α-d-Hep*p*4*P*-OMe (**C3**), l-α-d-Hep*p*4*P*-(1→3)-l-α-d-Hep*p*4*P*-OMe (**C4**) and l-α-d-Hep*p*-(1→7)-l-α-d-Hep*p*4*P*-OMe (**C5**). Focusing on the interface between the inner core and the Lipid A of LPS, the above-mentioned disaccharide structures were extended, generating additional ligands of library C: l-α-d-Hep*p*-(1→7)-l-α-d-Hep*p*4*P*-(1→3)-l-α-d-Hep*p*4*P*-(1→4)-α-Kdo*p* (**C6**), l-α-d-Hep*p*-(1→7)-l-α-d-Hep*p*-(1→3)-l-α-d-Hep*p*4*P*-(1→4)-α-Kdo*p* (**C7**), l-α-d-Hep*p*-(1→7)-l-α-d-Hep*p*4*P*-(1→3)-l-α-d-Hep*p*-(1→4)-α-Kdo*p* (**C8**), l-α-d-Hep*p*-(1→7)-l-α-d-Hep*p*-(1→3)-l-α-d-Hep*p*-(1→4)-α-Kdo*p* (**C9**). Compounds **C1–C9** are shown in Figure S4. Starting from the structure of the tetrasaccharide **C6**, the oligosaccharide structure was extended at the reducing end residue to give the following ligands: l-α-d-Hep*p*-(1→7)-l-α-d-Hep*p*4*P*-(1→3)-l-α-d-Hep*p*4*P*-(1→5)-[α-Kdo*p*(2→4)]-α-Kdo*p* (**C10**), l-α-d-Hep*p*-(1→7)-l-α-d-Hep*p*4*P*-(1→3)-l-α-d-Hep*p*4*P*-(1→5)-[α-Kdo*p*(2→4)]-α-Kdo*p*-OMe (**C11**), l-α-d-Hep*p*-(1→7)-l-α-d-Hep*p*4*P*-(1→3)-l-α-d-Hep*p*4*P*-(1→5)-[α-Kdo*p*(2→4)]-α-Kdo*p*-(2→6)-β-d-Glc*p*NAc-OMe (**C12**). Compounds **C10–C12** are presented in Figure S5.

### 2.2. Molecular Docking

**Figure 4:**
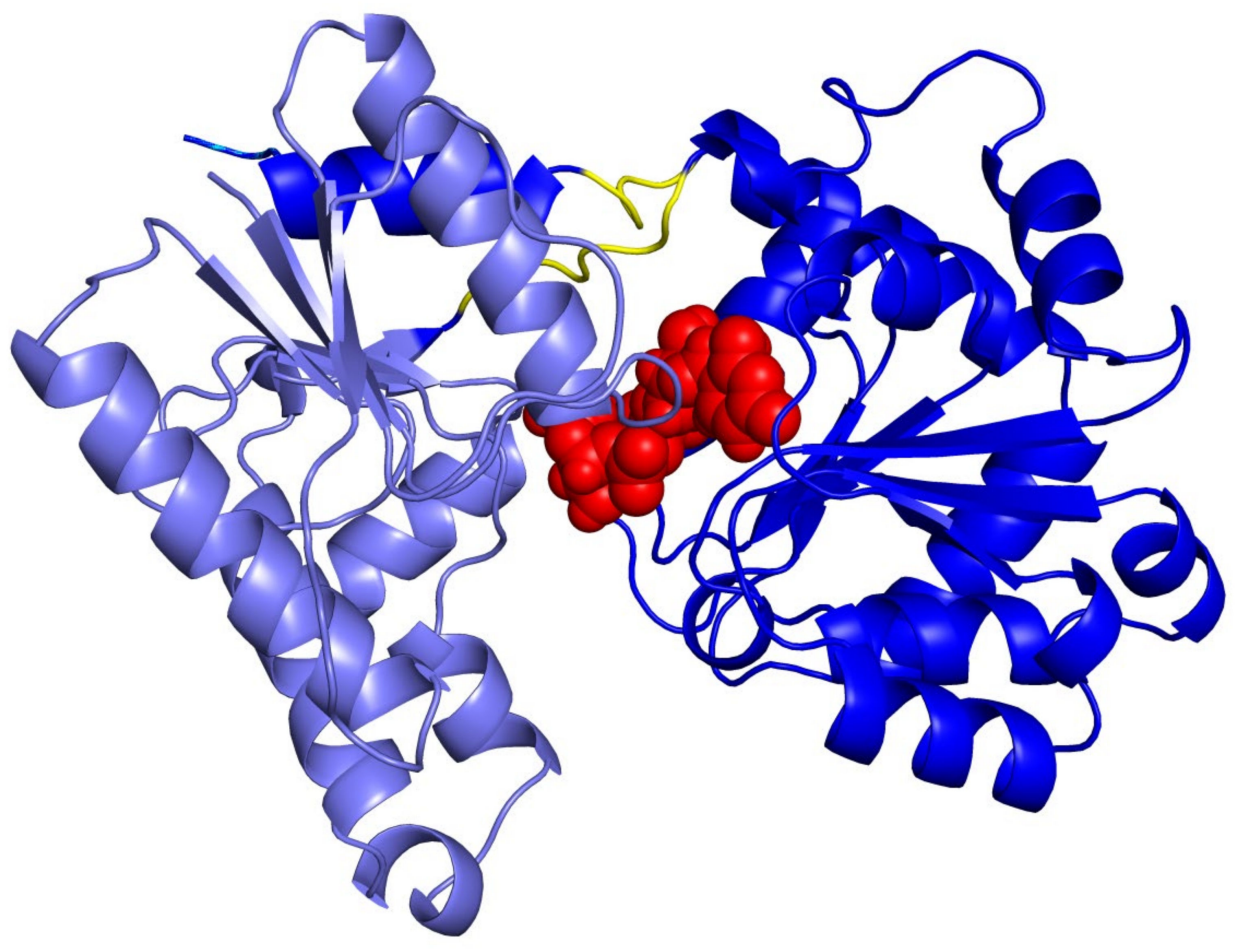
Conformation of UDP-Glc^2−^ in the binding pocket of WaaG, derived from U2F deposited co-crystallized structure (PDB ID: 2iw1).

Four docking programs, viz., AutoDock Vina [30], LeDock [31], rDock [32] and GOLD [33], were selected based on their sampling and scoring functions [34]. The different algorithms and approaches led to similar binding poses for the ligands. However, the predicted order of poses was usually different in terms of affinity energy. Each docking program generated the top energy-ranked binding poses for ligands of libraries A, B and C. The ranked affinity energies and scores are reported in Table S2. By comparing the binding mode of the natural ligand UDP-Glc^2−^ (Figure 4) derived from the co-crystallized structure of U2F, in the 2iw1 structure and the best-predicted poses of ligands with higher affinity, it was possible to see if the ligands bind preferably in the uridine sub-pocket, in the diphosphate region or in another portion of the binding pocket. Each ligand was docked in two different environments: (i) without any donor-related structure in the binding pocket of WaaG (i.e., the *apo*-form of protein) and (ii) having UDP-Glc^2−^ in the active site. The analysis of the results for the docking studies with the *apo*-protein was made considering the interactions of each ligand with the binding pocket of WaaG.

The first docking simulations involved the *apo*-form of WaaG and the ligands of the three libraries. The top-scoring hits of library A showed AutoDock Vina affinity energies that ranged from −6.6 to −8.7 kcal·mol^−1^, comparable to the binding free energies of fragments investigated in the design of norovirus inhibitors [35].

*Docking with WaaG* apo-*form: library A.* In the best binding pose of **A1**, the ligand occupies the uridine sub-pocket. The HO3′ of the ribosyl residue in UDP-Glc^2−^ interacts with the carboxylic group of Glu289 present in the binding pocket of WaaG [36]. In the same way, the amino group and the nitrogen atom of the thiazole in compound **A1** make H-bonds with the same carboxyl group of Glu289. Compound **A7** sits instead in the UDP-Glc^2−^ uridine-diphosphate portion of the binding pocket. The nitrogen of the thiazole ring in **A7** receives an H-bond from the NH_3_^+^ group of Lys209, while the amino group is the donor of an H-bond with the carboxylate group of Glu281. Thus, the phenyl-aminothiazole backbone is favorable for binding in the donor pocket of WaaG. The phenylthiazole scaffold of **A9** overlaps with the uridine residue of UDP-Glc^2−^, but the thiazole ring is oriented differently. In its best-predicted pose, ligand **A9** interacts with its thiazole nitrogen with the guanidino NH_2_ of Arg173, while the amino group of **A9** donates an H-bond to the carboxylate of Glu289. The top-ranked pose of compound **A14** has the ligand binding in the uridine portion of the donor pocket; in particular, the indole portion is oriented in the same way as the uracil ring, while the thiazole ring is in the ribose binding region. Compound **A16** binds in the same region as UDP-Glc^2−^, where its carboxyl group overlaps with the α-phosphate portion of UDP-Glc^2−^ (the phosphate group linked to the ribosyl residue). The carboxyl group on the phenyl ring plays a noteworthy role in the binding: it makes H-bonds with Ile285 and Val286, known to interact with the α-phosphate group of UDP-Glc^2−^. The hydroxyl group of Ser204 acts as a donor for a hydrogen bond to the oxygen atom of the aldehyde group on the pyrrole ring in **A16**.

*Docking with WaaG* apo-*form: library B.* The top-scoring ligands of library B ranked their AutoDock Vina affinity energies from −7.0 to −8.8 kcal·mol^−1^. Compound **B3d** has a favorable binding conformation for this stereoisomer. The other high scoring ligands **B16**, **B22**, **B26** and **B33** bind in the same UDP-Glc^2−^ pocket region. The benzimidazole moiety of compound **B16** is positioned in the UDP-Glc^2−^ ribose site, and the thiadiazole ring is in the β-phosphate region, while the phenyl portion is in the UDP-Glc^2−^ glucose region. The nitrogen atoms of the thiadiazole ring in **B16** play an important role: they act as acceptors of polar interactions both with the NH_3_^+^ group of Lys209 and with the guanidine NH_2_ group of Arg208. Compounds **B22** and **B33** bind in the uridine binding pocket, and the O-phenyl moiety of **B22** occupies the uracil portion in the binding pocket, while the ester chain overlaps with the phosphate-region of UDP-Glc^2−^. The carbonyl group of the ester group points in the same direction as a P=O group of UDP-Glc^2−^. Compound **B26** mainly occupies the UDP-Glc^2−^ binding region and points out of the pocket with one of the methylphenoxy groups. As for **B16**, both thiadiazole nitrogen atoms of compound **B26** interact with the donor NH_3_^+^ group of Lys209, while the sulfonamide group accepts two H-bonds from Gly15 and Leu16. The thiadiazole nitrogen of **B33** interacts with the amino group of Val286 in the UDP-Glc^2−^ pocket region. The lateral sulfonamide NH hydrogen atom makes H-bonds with the carboxyl group of Glu281. Each of the sulfonyl oxygens in **B33** interacts with the NH hydrogen atoms of Ala283 and Ile285.

**Figure 5:**
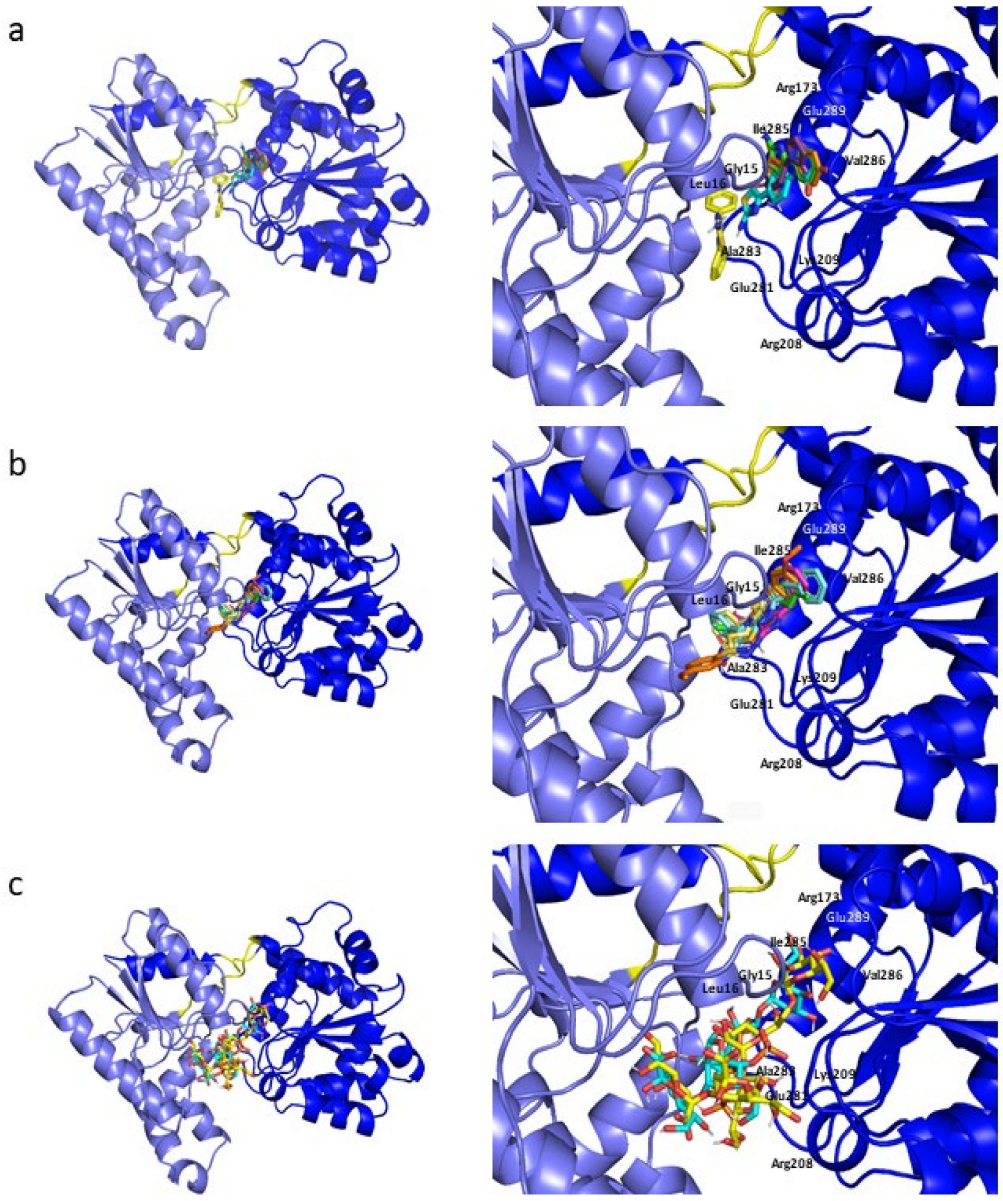
Docking poses of the representative ligands of libraries (**a**) A, (**b**) B and (**c**) C. On the right, a close-up of WaaG binding pocket showing the most relevant amino acids surrounding the ligands structures. (**a**) compound **A1** in green, **A7** in blue marine, **A9** in yellow, **A14** in orange and **A16** in violet. (**b**) compound **B3d** in blue marine, **B16** in light yellow, **B22** in fuchsia, **B26** in orange and **B33** in green. (**c**) compound **C6** in blue marine and **C10** in yellow.

*Docking with WaaG* apo-*form: library C.* By focusing on WaaG *apo*-protein, each docked pose of oligosaccharides **C1–C12** was analyzed for its interaction with the exposed amino acids within the binding pocket. The ligands of library C generally bind in the outer portion of the binding pocket of WaaG, where some overlap corresponds to the region of the β-phosphate group of UDP-Glc^2−^. The most interesting ligands from library C were **C6** and **C10**. Tetrasaccharide **C6** docked in the binding pocket with its non-reducing end heptose (Hep-III), whereas the Kdo residue is pointing out of the pocket. Hep-III overlaps with the ribose portion of UDP-Glc^2−^—in particular, the hydroxymethyl group of the ribose moiety and the pyranose ring of Hep-III in **C6**. Regarding the best binding poses of **C10**, the two Kdo residues point out of the pocket, while the Hep-III portion almost overlaps with UDP-Glc^2−^ ribose moiety. Furthermore, the Hep-II phosphate group binds in the region of the UDP-Glc^2−^ β-phosphate. A selection of representative ligands is reported in their best-docked poses in Figure 5a–c. Docking studies in the presence of WaaG-UDP-Glc^2−^ complex are discussed in some detail in the Supporting Information. Ligands with higher affinity for their best-predicted pose were chosen and subjected to molecular dynamics (MD) simulations.

### 2.3. Molecular Dynamics Simulations

In order to better understand the interactions between WaaG and its natural substrates and to further extend the results from docking studies of libraries A, B and C, 10-ns molecular dynamics (MD) simulations were carried out on both ligands and protein-ligand complexes. For library A, ligands **A4**, **A8** and **A15** were selected via STD NMR experiments (*vide infra*), exhibiting a good protein-ligand affinity profile. Docking studies (in the presence of WaaG *apo*-form or WaaG-UDP-Glc^2−^ complex) allowed a selection of ligands with the highest affinity, to be further investigated with MD simulations. Only the top-ranked ligands from each library were considered for the MD calculations. For the selected ligands, of the ten binding poses generated by each run for every ligand, the top-predicted binding pose was utilized to generate the initial coordinates for the MD trajectories. Top-ranked binding conformations of the disaccharides **C1**, **C2**, **C3** and **C4** from library C were prepared for molecular dynamics investigations, considering, in particular, their protonation states. In this regard, phosphate groups in position 4 of ligands **C3** and **C4** were considered in a protonated form (**C3** and **C4**) or a monocharged form (**C3**^1−^) or were doubly charged (**C4**^2−^). Explicit solvent MD calculations were carried out, solvating each ligand and protein-ligand complex in a 50 Å square water box neutralized with NaCl. The default time step was 2 fs at 310 K and 1 atm; timesteps of 1 fs or 0.1 fs were utilized in the case of unstable trajectories when a larger timestep was used.

### 2.4. Binding Free Energy (BFE) Calculations

Molecular dynamics output (trajectories, DCD files) of the selected ligands were first subjected to binding free energy (BFE) analysis using Linear Interaction Energy (LIE) methods [37,38]. Table 1 reports the variation in Gibbs Free Energy of Binding (Δ*G*_bind_) expressed in kcal·mol^−1^ and the related dissociation constant (*K*_D_) in mM for each WaaG/ligand complex. The results are reported in descending order, in each of the three parts of the table. For library A, ligands **A7** and **A4** have the most negative values of BFE, giving *K*_D_s slightly lower than mM. Compound **A9** has a *K*_D_ value in the order of mM, while ligands **A15**, **A14** and **A1** have only slightly negative values of BFE. Compounds **A8** and **A16** seem not to have a good binding profile to WaaG, having unfavorable (positive) values of BFE. Ligands from library B with bigger molecules clearly have a stronger affinity for the exposed amino acids in the binding pocket of WaaG. Ligand **B33** is predicted to have the best affinity for the active site for this library, showing a *K*_D_ value of 40 µM. Derivatives **B16** and **B22** have *K*_D_s in the mM range, showing similar Δ*G*_bind_ values, while **B3d** and **B26** both have larger *K*_D_ values, binding less tightly than the other ligands of the same library. Regarding the disaccharide ligands of library C, compound **C1** has a *K*_D_ value in the mM range, and so does **C3**. Its mono-charged form, **C3^1^**^−^, seems to be a weaker binder compared to ligand **C3**. The mono phosphorylation on the reducing end heptose in **C3** seems not to play an important role for the binding with WaaG, since compound **C2** is a stronger binder, with a more negative value of Δ*G*_bind_ and a *K*_D_ value of 70 µM. On the other hand, the double phosphorylation on the two heptose residues in **C4** is beneficial for the binding. Both the protonated (**C4**) and the doubly charged (**C4^2−^**) forms of the ligand have *K*_D_ values in the µM range—0.8 and 0.4 µM, respectively.

### 2.5. RMSD Analysis of WaaG Backbone

**Figure 6:**
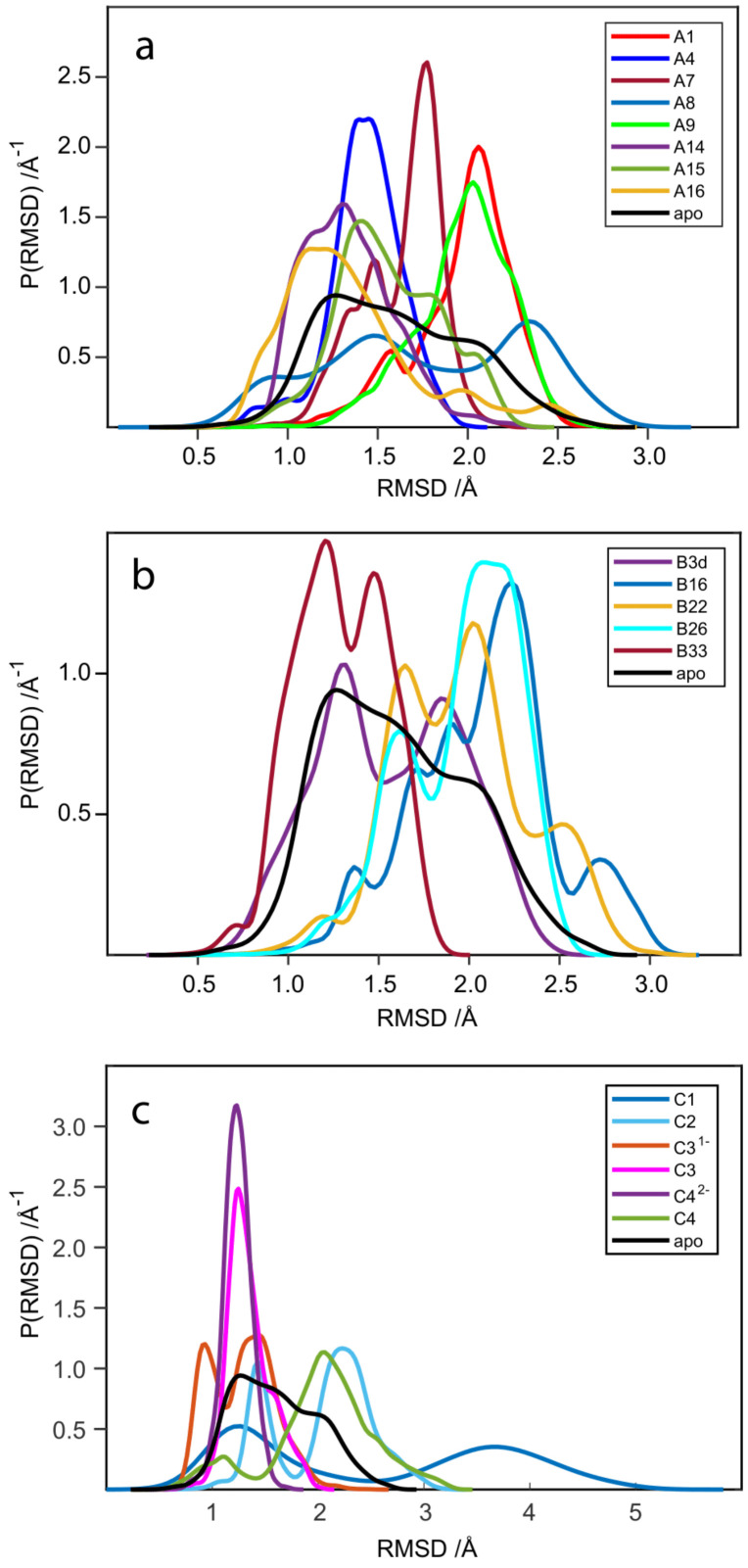
RMSD probability distributions obtained by a kernel density estimate using a normal distribution function. The calculation is done on the protein backbone of the *apo*-protein (from 2iw1) and the various WaaG-ligand complexes. The panels depict the RMSD distributions for WaaG in complex with ligands of (**a**) library A, (**b**) library B and (**c**) library C.

Analysis of the root-mean-square-deviation (RMSD) distribution of WaaG *apo*-protein backbone and the various WaaG-ligand complexes was considered as a tool to study the dynamics of each system (Figure 6). The calculation refers to WaaG backbone spatial changes during the 10-ns MD production. For the three libraries, it is evident that the flexibility of the protein backbone in the different complexes can vary in proportion to the active site adaptation to the ligand conformational state. Figure S6 depicts the RMSD as a function of the duration of the MD simulation. In addition, Table S3 in Supporting Information reports the average RMSD value and the related standard deviation (SD). The avRMSD value for the reference *apo*-form of WaaG is 1.31 ± 0.16. For the RMSD analysis in Figure 6a–c, the WaaG *apo*-form is reported as “apo” in black as a reference RMSD distribution. Regarding library A, the protein backbone for the complex WaaG-**A8** is quite flexible, defining a broad distribution of **A8** for the WaaG-binding pocket in terms of BFE and *K*_D_ values. On the other hand, compound **A16** (which was recognized as a weak binder) gives more stability to WaaG backbone compared to **A8**, even with a slightly broad main distribution. In complex with compound **A15**, the protein backbone showed three dynamic states ranging from 1.0 to 1.5 Å of RMSD variation. When bound to compounds **A7** or **A14**, WaaG backbone presents a narrow distribution of RMSD, besides a low-populated state at the initial portion of the production phase. Ligands **A1** and **A9**, when bound to WaaG, result in a comparable curve with a broad profile, whereas the complex WaaG-**A4** generates a narrow ΔRMSD distribution of WaaG backbone, with an avRMSD of 1.43 Å ± 0.21. Regarding library B, there is a trend of increased instability of WaaG backbone in comparison to the ligands of library A. In general, library B ligands generate two main ΔRMSD distributions of WaaG *apo*-protein. The more stable complex is represented by WaaG-**B33**, with a range of RMSD variation of 1.5–2.0 Å and an avRMSD of 1.29 Å ± 0.25. This is also in accordance with the best BFE value of **B33** for library B. Regarding library C, disaccharide **C1** gives two main conformations of WaaG when bound to it, but each with a very broad RMSD variation, ~2.5 Å and a total range of > 5 Å of ΔRMSD. Ligand **C2** generates two well-distinguishable and comparably populated states of the protein backbone. Disaccharide **C4**^2−^ with the best affinity and best *K*_D_ value, as seen before, gives great stability to WaaG backbone, with a small range of RMSD variation (~0.5 Å), very similar to the distribution of RMSD for the protein backbone when **C3** is bound to it. The better stability of **C3** compared to **C3**^1−^ is in accordance with the better affinity in terms of *K*_D_ values of **C3** compared to **C3**^1−^. The charged compound **C3**^1−^ generates two equally populated states of the WaaG backbone; on the other hand, WaaG has a conformational state when bound to disaccharide **C4**, presenting a quite broad RMSD distribution similar to that of the protein conformation in complex with **C2**.

### 2.6. Electrostatic and van der Waals Contributions to WaaG/Ligand Binding

**Figure 7:**
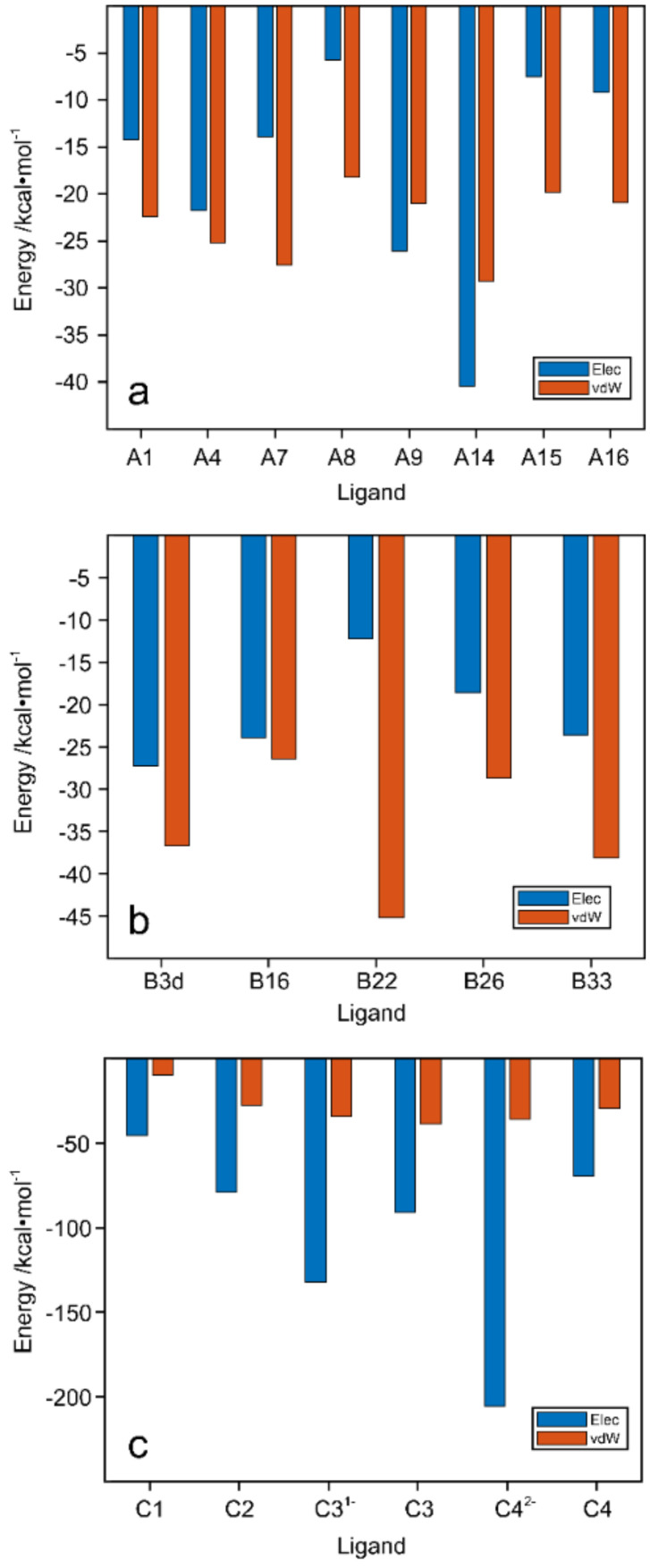
Contributions of electrostatic and van der Waals interactions to the binding of (**a**) library A, (**b**) library B and (**c**) library C ligands to WaaG binding pocket. Elec = electrostatic; vdW = van der Waals.

Figure 7a–c shows a histogram plot of the difference in polar and nonpolar interaction between each ligand and WaaG-binding cavity. In general, ligands of library A have the van der Waals (vdW) term higher than the electrostatic one for the binding affinity. Only ligands **A9** and **A14** showed a slightly higher electrostatic term compared to the vdW one. Additionally, for the ligands of library B, the vdW term is predominant, particularly for ligand **B22**. An inverse trend is displayed for the disaccharides of library C, where the electrostatic term is higher due to the presence of numerous hydroxyl groups on the disaccharides and because of their interactions with polar portions of the amino acids of the binding pocket. The electrostatic term is even more evident for the phosphate-charged disaccharides **C3**^1−^ and **C4**^2−^, similar to the importance of the electrostatic term in the interaction between negatively charged oligosaccharides and the Aβ42 peptide [39].

### 2.7. Influence of the H-Bonds for WaaG/Ligand Binding Affinity

**Figure 8:**
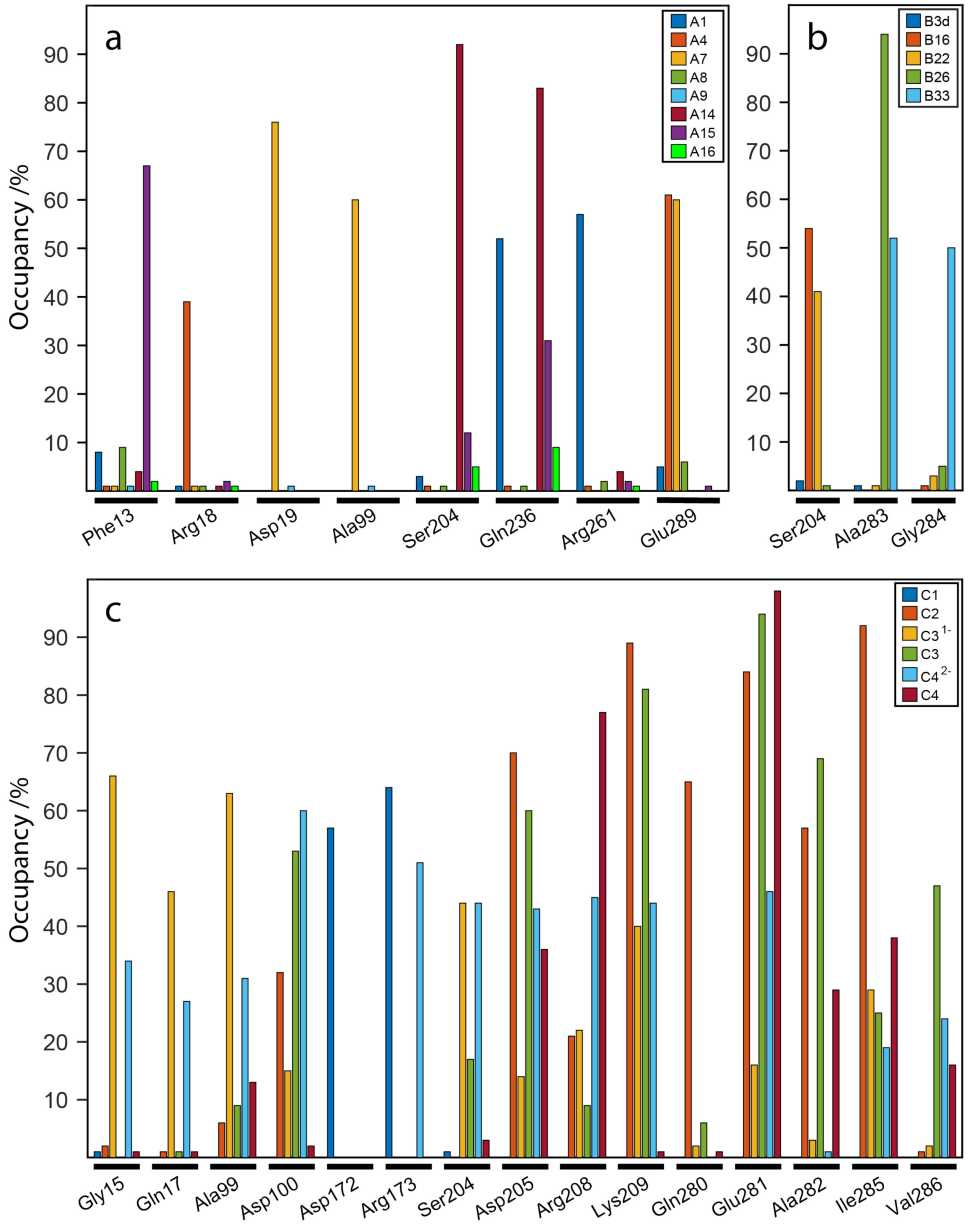
Hydrogen bond occupancies for ligands of (**a**) library A, (**b**) library B and (**c**) library C to the amino acids in the binding pocket of WaaG. Reported amino acids have at least 30% occupancy for one protein/ligand H-bond.

Figure 8a–c presents a series of histograms with different colors representing different ligands binding the active site of WaaG. In the *x*-axis, there are reported amino acids of the binding pocket which are representative for the polar interactions of those ligands (i.e., having at least an H-bond with 30% occupancy for at least one ligand). The occupancy in the *y*-axis is reported in percentage and is estimated taking into account the presence of that particular H-bond during the 10-ns MD calculation. This analysis shows compounds **A8** and **A16** as weak binders because they do not have H-bonds with any notable occupancy during the 10-ns calculation. Phe13 has a selective H-bond for **A15** and likewise for Arg18, which has a polar contact with ligand **A4**, which, in turn, also binds with Glu289. Compound **A7** is confirmed to be a good binder, with two selective H-bonds with Asp19 and Ala99 but also an H-bond with Glu289, similarly to **A4**. Compound **A1** has two representative H-bonds with Gln236 and Arg261, while **A14** binds with Ser204 and Gln236 with a high occupancy (85–90%). As for library B, ligand **B33** has two H-bonds with Ala283 and Gly284 with a 50% occupancy. Compound **B26** has a >90% occupancy for an H-bond with Ala283. Ser204 has two H-bonds with 40–55% of occupancy with ligands **B16** and **B22**. Library C, as seen above, has a higher electrostatic term for its ligands, which is reflected in the great number of H-bonds between these disaccharides and the exposed amino acids of the binding pocket. Disaccharide **C1** has a selectivity for the hydrogen bonds with Asp172 and Arg173, with a 60–65% of occupancy. In addition, there is a marked difference between compounds **C1** and **C2** in terms of H-bonds. In support of this, disaccharide **C2** has different H-bonds with different occupancies. The most representative ones are with Asp205, Lys209, Gln280, Glu281 and Ile285, with occupancies between 70 and 90%. Disaccharide **C3**^1−^ has two important H-bonds, with 65% occupancy, with Gly15 and Ala99. Compound **C3** has several H-bonds, but the most important are with Lys209 and Glu281. The doubly charged ligand **C4**^2−^ has two important H-bonds with Asp100 and Arg173 and also ~10 polar bonds with lower occupancy (~30–40%). Disaccharide **C4** has a 75% occupancy for the H-bond with Arg208 and almost 100% of the MD simulation with Glu281.

### 2.8. Screening by NMR Spectroscopy

**Figure 9:**
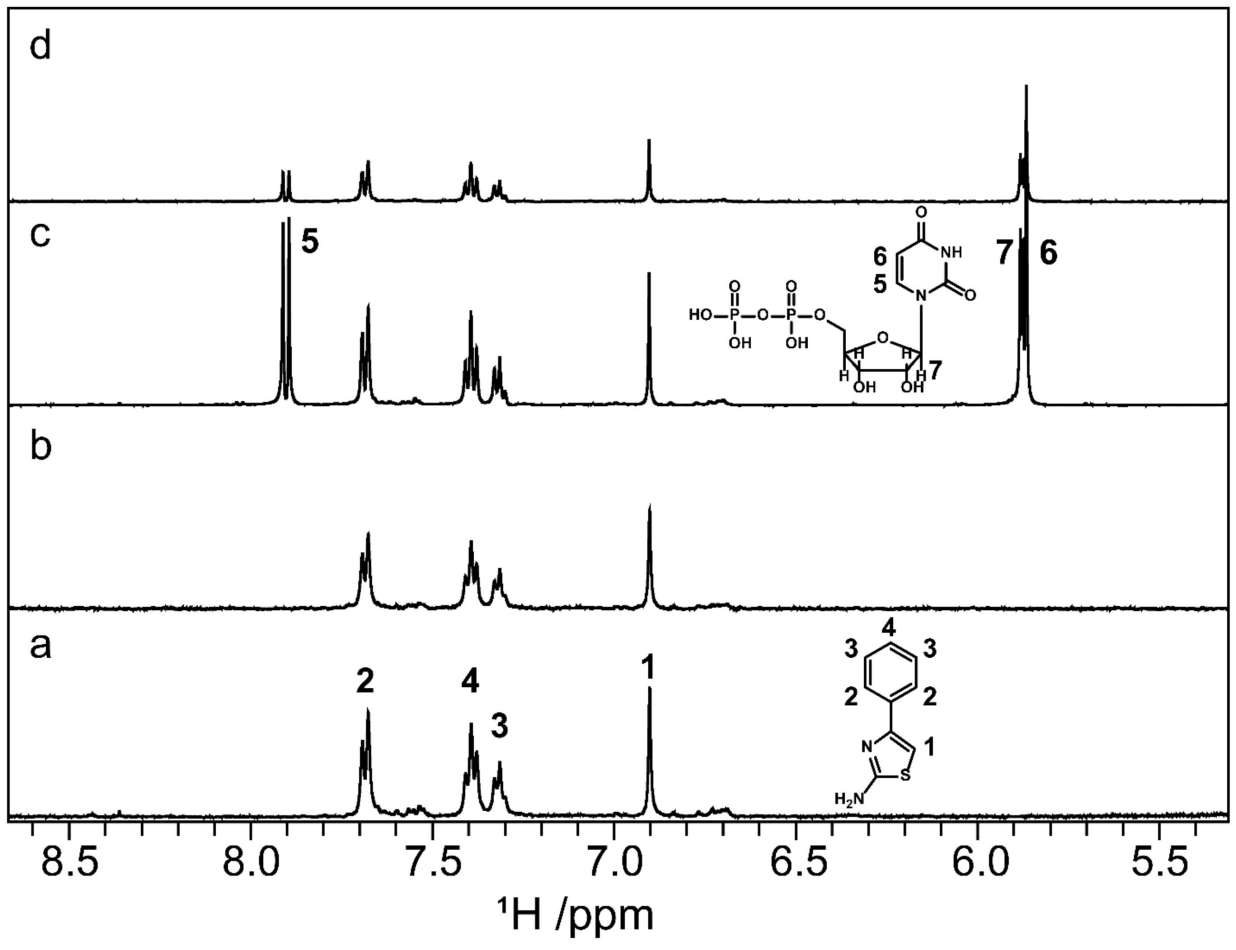
STD NMR spectra of ligand **A4** and WaaG carried out with and without UDP as a competitor. In sequence from the bottom to the top, 1D ^1^H STD off-resonance (**a**) and on-resonance (**b**) irradiation of WaaG in the presence of **A4** and off-resonance (**c**) and on-resonance (**d**) irradiation with UDP added to the protein-ligand mixture. Compound **A4** shows interaction with WaaG also in the presence of UDP.

Saturation Transfer Difference (STD) NMR experiments [27] were performed on different ligands of library A and disaccharides **C1**, **C2**, **C3** and **C5** of library C. Library A showed significant STD effects for three of the ligands analyzed: compounds **A4** (Figure 9), **A8** and **A15**, which was also the case for **A4** in conjunction with UDP. The STD effects were uniformly distributed for the protons throughout the **A4** molecule, and a similar pattern was observed in the binding of **A15** together with UDP and WaaG. Several other compounds from library A gave less than 1% of STD signal and were disregarded for further evaluation. Compounds of library B were not analyzed in this study via STD NMR. The oligosaccharides analyzed from library C showed only weak STD signals (less than 1%) and were therefore not analyzed further. As small saccharides generally are weak binders [40] and the STD NMR technique detects binding to proteins for ligands with *K*_D_ ranging from mM to μM or even lower [41], we conclude that the absence of notable interactions between the disaccharides and WaaG indicates that the minimal motif needed for recognition of the oligosaccharide acceptor has not been fulfilled and that a larger oligosaccharide structure is required for the glycosylation reaction to take place.

### 2.9. WaaG Has a Twisting-like Dynamics Involving N- and C-Domains

**Figure 10:**
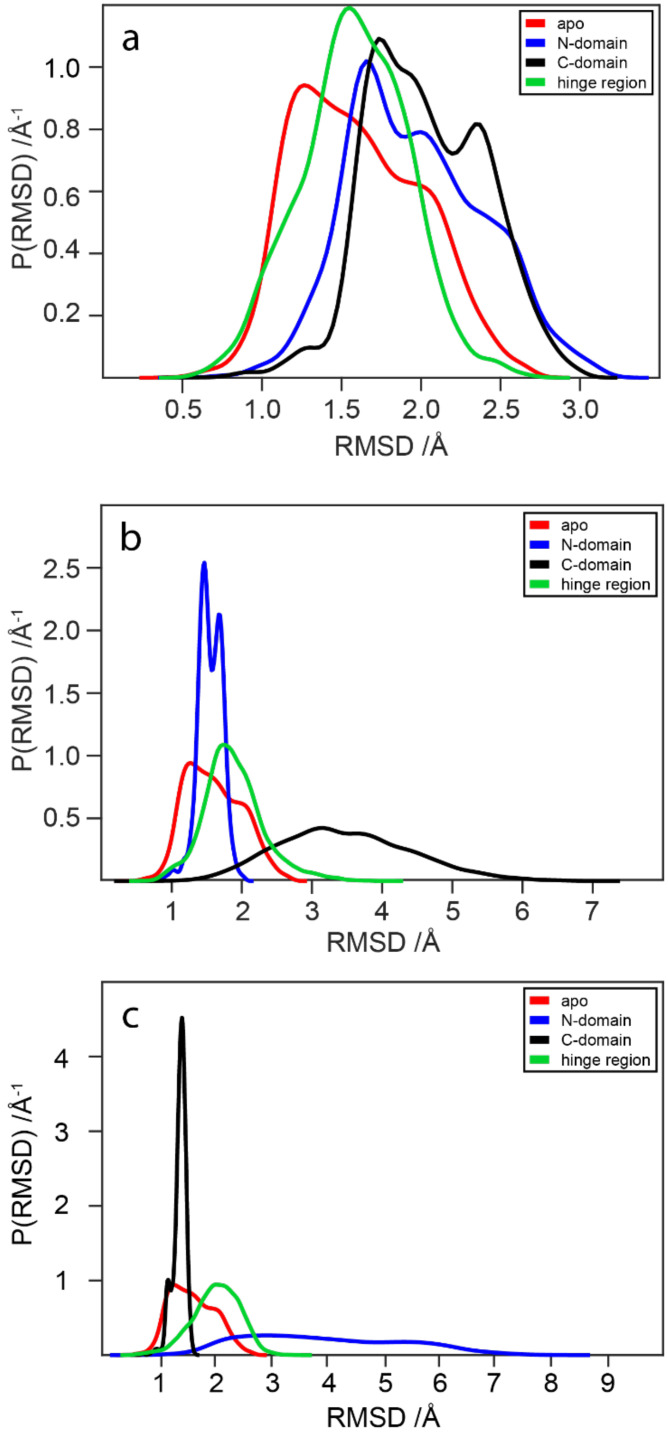
RMSD probability distributions obtained by a kernel density estimate using a normal distribution function. The calculation is done on the protein backbone of the *apo*-protein (from 2iw1, red) as a reference of RMSD values. Alignment of (**a**) the entire protein backbone, (**b**) N-domain, and (**c**) C-domain of *apo*, then RMSD calculation of the N- and C-domain and hinge region.

WaaG is a protein constituted by 371 amino acids and has two well-distinguishable domains, the N- and C-domains. The N-domain is recognizable between the amino acids Met1 and Gln163; a central part, denoted as the “hinge” region (amino acids from Ile164 to Pro171), then links the N- to the C-domain. The latter constitutes amino acids between Asp172 and Gly371, considering that there is another “hinge” portion (Gln355-Tyr358), and the C-terminus helix (Ser359-Gly371), even if the last residues are spatially in the N-domain. The starting point is the calculation of the RMSD variation for the *apo*-protein WaaG during the 10-ns production. In Figure 10a–c, the red curve represents the reference RMSD distribution of WaaG *apo*-protein. Focusing only on a particular portion of the protein, the blue, black and green curves refer to the RMSD values for the N-, C-domain and the hinge region, respectively. In Figure 10a, there is only a slight difference between the reference (*apo*) and the different domains of the protein of the RMSD distribution. In Figure 10b,c, the alignment is made on the N- and C-domain, respectively. When the alignment is done on a specific domain, it is possible to observe that the opposite domain presents a broad distribution of the backbone RMSD, which signifies the dynamic behavior of the two domains that move relative to each other.

**Figure 11:**
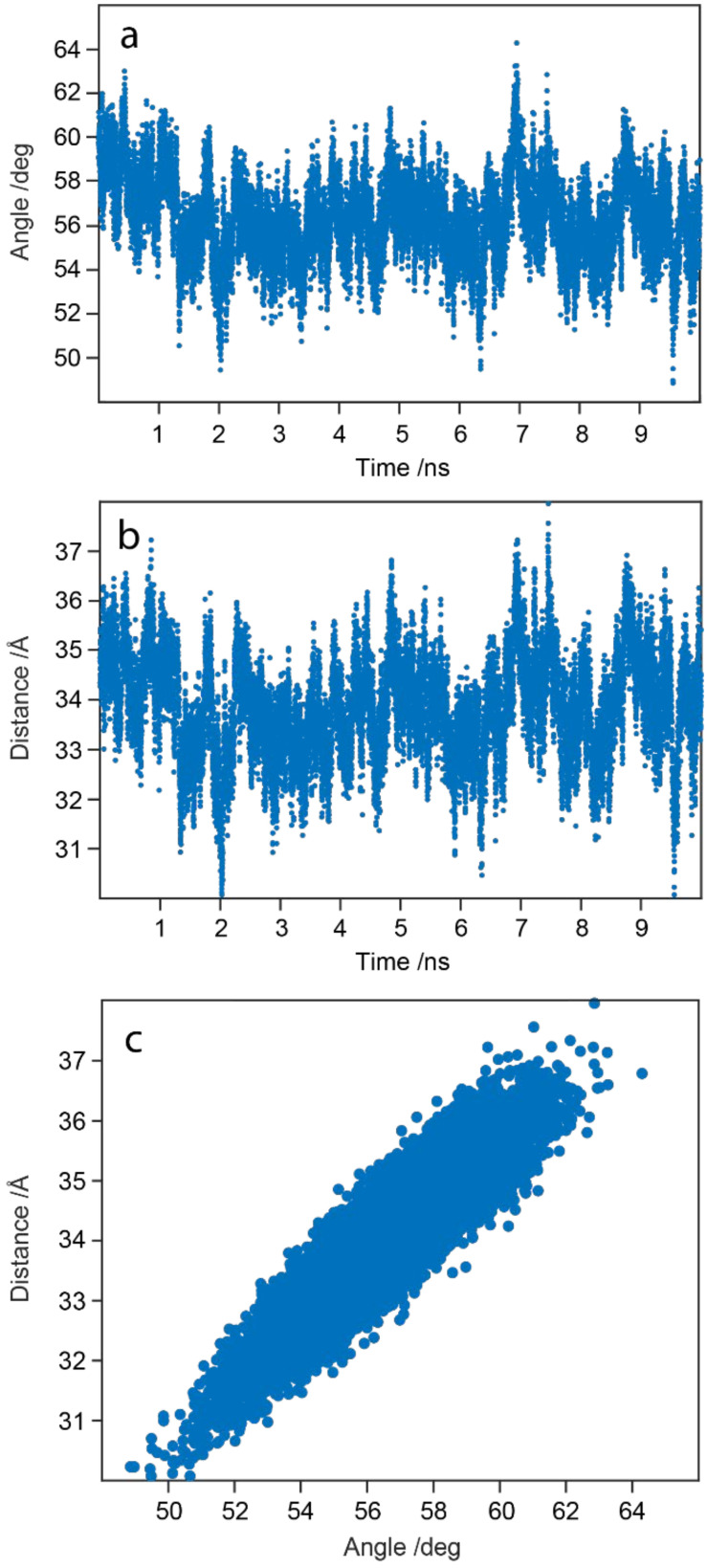
(**a**) Angle variation over time between the Cα atoms of His62 (N-domain), Lys248 (C-domain) and Gly168 (hinge region) and (**b**) distance fluctuations over time between the Cα atoms of His62 and Lys248 during the 10-ns MD calculation. (**c**) Scatter plot of the two variables against each other.

**Figure 12:**
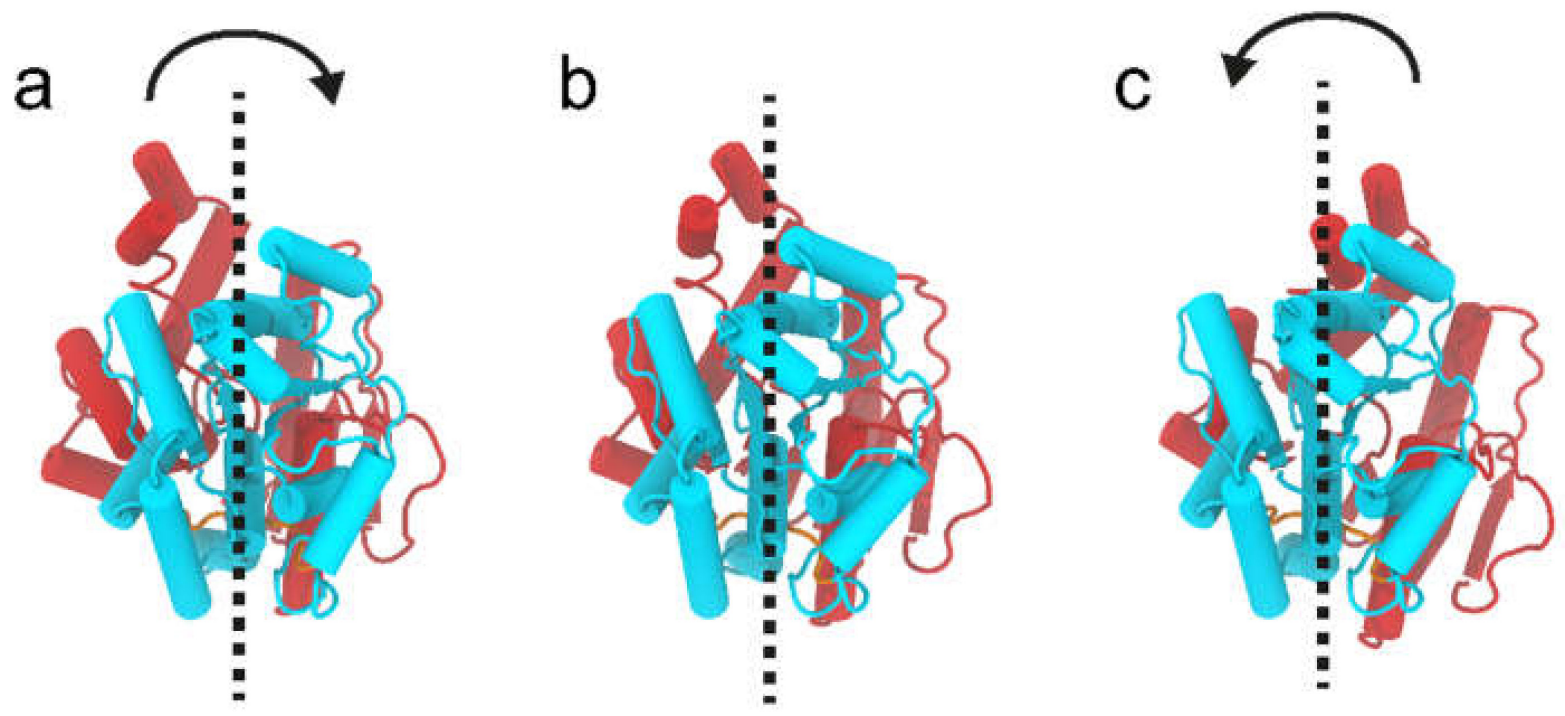
Graphical representation of the twist-like dynamics of WaaG. The C-domain of the protein (in cyan) is kept aligned, while the rest of the protein moves. Depicted are the two extremes (**a**,**c**) of the twist-like movements of the N-domain (in brick-red color) with respect to the C-domain and a representation of the two domains when aligned in the eclipsed state (**b**).

Figure 11a–c reports an additional analysis of the internal dynamics of WaaG protein over the MD trajectory. Figure 11a shows the variation over time of the angle defined between the Cα atoms of His62 in the N-domain, Lys248 in the C-domain and Gly168 in the hinge region. Figure 11b depicts the change in distance between the Cα atoms of His62 (N-domain) and Lys248 (C-domain). The variation of the angle and the distance presents a similar trend, suggesting a twisting-like movement (Figure 12). To confirm the correlation between these two variables, they were plotted together in Figure 11c, and the Spearman correlation coefficient *rho* was calculated. The correlation coefficient can have a range of values from −1 to +1, showing a perfect negative or positive correlation between the ranks of the two variables, respectively. The calculated Spearman’s *rho* is 0.9028, hence indicating a good correlation between these two variables.

### 2.10. In Vitro WaaG Inhibition Assay

The WaaG activity assay utilized the previously developed protocol [23] for testing compounds of libraries A and B. However, none of the fragment-based compounds (library A) or small molecules (library B) showed any significant inhibitory effect at a lower concentration than that previously determined for **A1**, which had a half-maximal inhibitory concentration (IC_50_) of 1 mM.

## 3. Discussion

**Figure 13:**
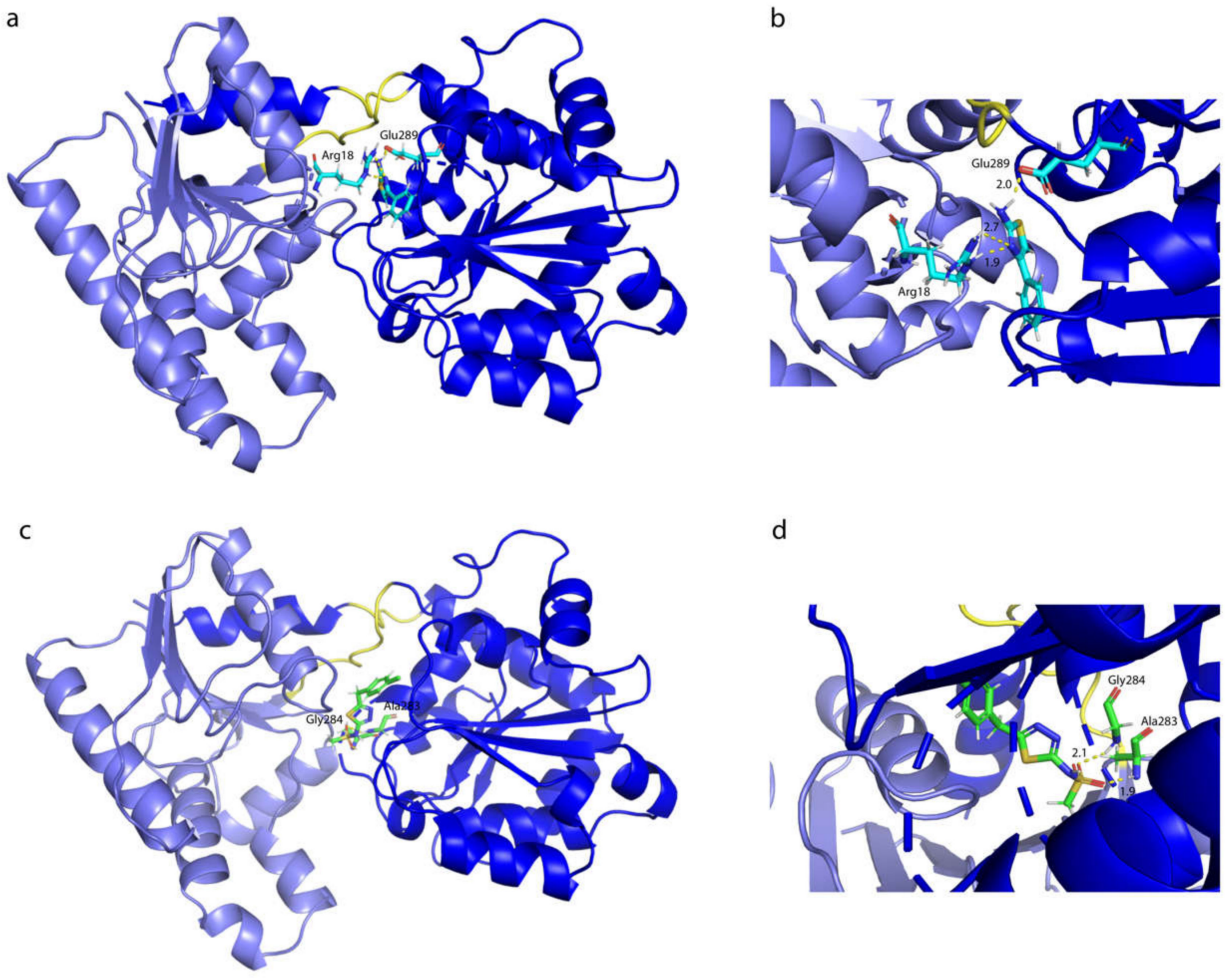
Molecular dynamics representation and interactions of ligands (**a**,**b**) **A4** and (**c**,**d**) **B33** with WaaG binding pocket. The structures are the most representative of the different clusters identified for each ligand.

The present work describes a combination of computational and experimental studies aimed at discovering new fragment molecules that could be identified as inhibitors for glucosyltransferase WaaG, but also at discovering new insights on the mechanism of action of the protein and the contact between the donor UDP-Glc^2−^ and the acceptor portion (l-*glycero*-d-*manno*-heptose-II) of LPS. Last but not least, part of this work focused on the elucidation of the dynamic behavior of the protein in the physiological environment. The background was a combination of preliminary docking, NMR spectroscopy and biochemical experiments, where compound **A1** showed some inhibitory activity towards WaaG. This evidence was the basis for further developments using a fragment-based approach to identify new molecules with the potential of increased inhibitory effects of WaaG. Library A and B were first tested with a molecular docking approach, acting as a filter and to progress only with the most interesting compounds, comparing the docking outputs with UDP-Glc^2−^ WaaG structure (obtained by the crystal structure of U2F, PDB ID 2iw1), thereby selecting for them the best-docked pose. Compounds **A1**, **A7**, **A9**, **A14**, **A16**, **B3d**, **B16**, **B22**, **B26** and **B33** from libraries A and B were selected based on docking binding affinity. Meanwhile, compounds **A4**, **A8** and **A15** were selected from an experimental study involving the STD NMR technique, where these ligands showed substantial STD effects with WaaG *apo*-protein, as well as in the presence of UDP for **A4**. The best-predicted poses of all of the above-mentioned ligands from libraries A and B were employed for further MD simulations. Analyzing the BFE and *K*_D_ values for each ligand for its binding to the active site of WaaG, compounds **A7**, **A4** and **B33** for libraries A and B displayed a promising profile of BFE, with *K*_D _< mM (0.11, 0.62 and 0.04 mM, respectively). In order to study the effects on the protein backbone in the WaaG/ligand complex during the 10-ns MD simulation, an analysis of the variation of RMSD distribution was assessed. For library A, compound **A4** is undoubtedly the one that gives more stability to WaaG backbone, also compared to the reference RMSD analysis on the *apo*-protein, with an avRMSD of 1.43 Å ± 0.21. Regarding library B, ligand **B33** generates a good stability profile to the protein backbone, with an avRMSD of 1.29 Å ± 0.25. Investigating in more detail the binding between the ligands and the exposed amino acids in the WaaG active site, an analysis on the relative contributions of electrostatic and van der Waals interactions showed that ligand **A4** carries out a balanced binding between polar and nonpolar interactions, with an average energy of ~25 kcal·mol^−1^ for both. Library B, on the contrary, has a higher electrostatic term compared to the hydrophobic one. The hydrogen bonds between the ligands and the binding pocket were subsequently elucidated. Compound **A4** has H-bonds with Arg18 and Glu289 and compound **B33** binds to Ala283 and Gly284. Focusing on ligands **A4** and **B33**, a cluster analysis on the entire 10-ns MD simulation was explored. The different clusters of poses of a ligand were generated considering the difference in RMSD, with a cutoff value of 1 Å. The binding of the most representative pose of the clusters for compounds **A4** and **B33** is depicted in Figure 13.

**Figure 14:**
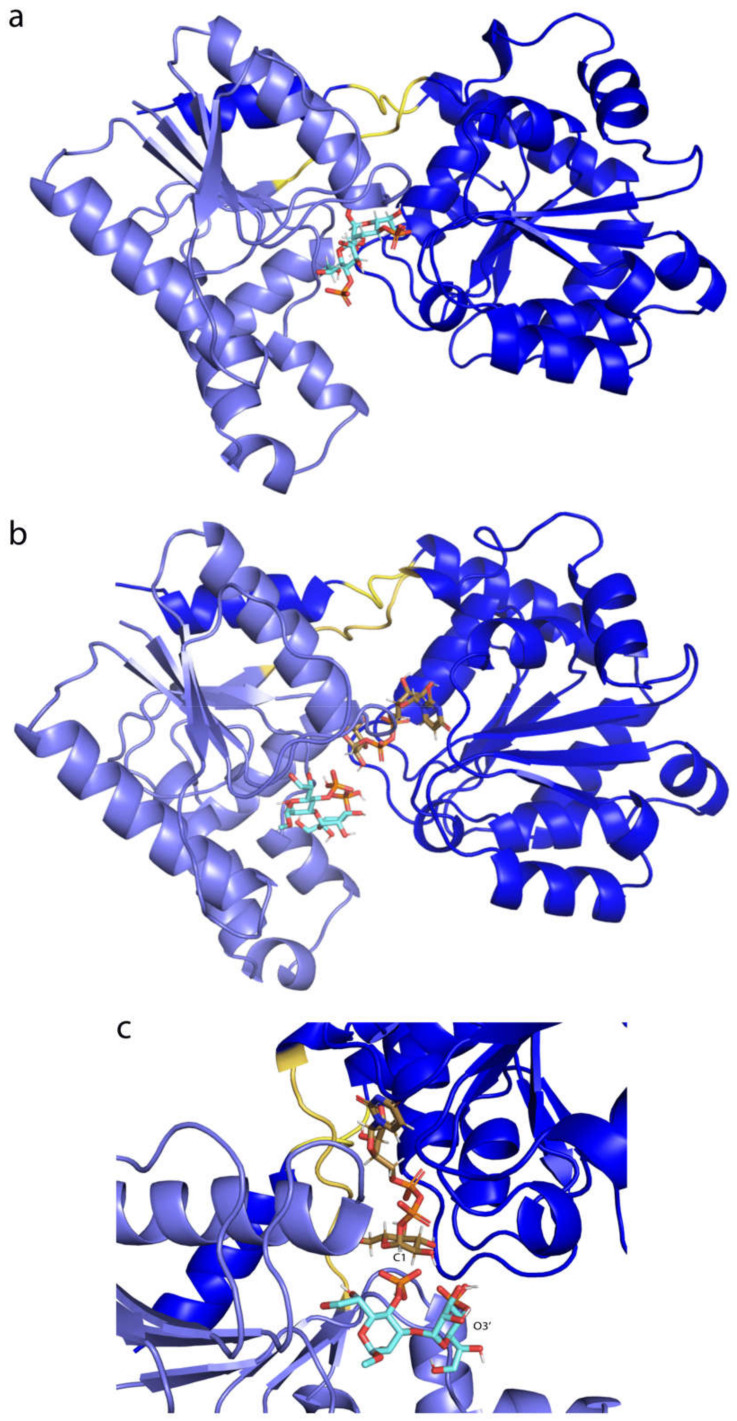
(**a**) Molecular dynamics representation of **C4**^2−^ and (**b**,**c**) molecular docking conformation of **C4**^2−^ with WaaG-UDP-Glc^2−^ complex.

In order to shed light on the mechanism of transfer of a glucose residue from UDP-Glc^2−^ (donor) to Hep-II (acceptor), ligands **C1**–**C12**, as models of different parts of the inner core of the LPS, were docked in the presence of *apo*-form, but particularly with WaaG-UDP-Glc^2−^. Only the disaccharides from libraries C, **C1**, **C2**, **C3** and **C4** were subjected to more detailed MD calculations because of the interest in investigating Hep-II, the acceptor of the monosaccharide residue from UDP-Glc^2−^. The phosphate-derived ligands were studied in both non-charged (**C3** and **C4**) and charged (**C3**^1−^ and **C4**^2−^) forms of the phosphate group(s). Doubly charged compound **C4**^2−^ has a *K*_D_ value in the µM range (0.4 µM), and the most representative conformation from the 10-ns MD simulation is depicted in Figure 14a. The ligands then displayed a preferred binding to the specific region of the WaaG active site where UDP-Glc is located. In contrast, the output of the docking calculation of **C4**^2−^ in the presence of UDP-Glc^2−^ in the binding pocket of WaaG results in the disaccharide being positioned in the vicinity of the glucosyl residue (Figure 14b,c).

## 4. Materials and Methods

### 4.1. General Experimental Methods

All reagents were used as delivered. The commercially available Dowex^®^ 1×4 chloride form, 50–100 mesh, strongly basic, was used to prepare the acetate form. TLC was carried out on silica gel 60 F254 and monitored with UV light at 254 nm. NMR spectra for the characterization of compounds **A11**–**A13** were recorded at 25 °C on a Bruker 600 MHz spectrometer. The NMR chemical shifts are reported in ppm and are referenced to the DMSO-*d*_6_ solvent peak at 39.52 ppm and the residual peak at 2.50 ppm for ^13^C and ^1^H, respectively. ^1^H NMR chemical shifts and *J*_H,H_ coupling constants were extracted from 1D ^1^H NMR spectra using the NMR spin simulation software PERCH [42,43]. Disaccharides **C1**, **C3** and **C5** were available from previous studies [44,45]. A selection of protein-ligand complexes in pdb-format is given as Supplementary material.

#### 4.1.1. 2-Acetamido-5-methyl-4-phenyl-1,3-thiazol (**A11**)

2-Amino-5-methyl-4-phenyl-1,3-thiazol (0.100 g, 0.53 mmol) and acetic anhydride (0.055 mL, 0.58 mmol) were dissolved in ethyl acetate (2 mL) and stirred overnight at rt. The formed white solid was filtered, washed with ethyl acetate and dried in vacuo to obtain the product as a white powder (100 mg, 82%). ^1^H NMR (DMSO-d_6_): δ 2.13 (s, 3H, MeCO), 2.45 (s, 3H, Me), 7.33 (dd, J_H-ortho,H-para_ 1.19 Hz, J_H-meta,H-para_ 7.41 Hz, 1H, H-para), 7.44 (dddd, J_H-ortho,H-meta_ 7.83 and 0.51 Hz, J_H-meta,H-meta_ 1.28 Hz, J_H-meta,H-para_ 7.41 Hz, 2H, H-meta), 7.63 (dddd, J_H-ortho,H-ortho_ 1.74 Hz, J_H-ortho,H-meta_ 7.83 and 0.51 Hz, J_H-ortho,H-para_ 1.19 Hz, 2H, H-ortho) (Figure S7). ^13^C NMR (DMSO-d_6_): δ 11.82 (Me), 22.40 (MeCO), 120.74 (C5), 127.17 (C-para), 127.92 (C-ortho), 128.34 (C-meta), 134.95 (C-ipso), 143.92 (C4), 153.96 (C2), 168.30 (CO) (Figure S8).

#### 4.1.2. 2-Formamido-5-methyl-4-phenyl-1,3-thiazol (**A12**)

2-Amino-5-methyl-4-phenyl-1,3-thiazol (0.100 g, 0.53 mmol) was dissolved in 1 mL freshly prepared acetic formic anhydride and stirred overnight at rt. The solvent was evaporated and the residue was washed with ethyl acetate to obtain the product as a white powder (96 mg, 83%). ^1^H NMR (DMSO-d_6_): δ 2.47 (s, 3H, Me), 7.34 (dd, J_H-ortho,H-para_ 1.22 Hz, J_H-meta,H-para_ 7.42 Hz, 1H, H-para), 7.44 (dddd, J_H-ortho,H-meta_ 7.81 and 0.58 Hz, J_H-meta,H-meta_ 1.41 Hz, J_H-meta,H-para_ 7.42 Hz, 2H, H-meta), 7.63 (dddd, J_H-ortho,H-ortho_ 1.86 Hz, J_H-ortho,H-meta_ 7.81 and 0.58 Hz, J_H-ortho,H-para_ 1.22 Hz, 2H, H-ortho), 8.47 (s, 1H, H_Fo_), 12.21, s, 1H, NH) (Figure S9). ^13^C NMR (DMSO-d_6_): δ 11.84 (Me), 121.55 (C5), 127.30 (C-para), 128.00 (C-ortho), 128.36 (C-meta), 134.72 (C-ipso), 144.23 (C4), 152.26 (C2), 159.43 (CO) (Figure S10).

#### 4.1.3. 2-Amino-3,5-dimethyl-4-phenyl-1,3-thiazolium acetate (**A13**)

2-Amino-5-methyl-4-phenyl-1,3-thiazol (0.100 g, 0.53 mmol) was dissolved in acetone, methyl iodide (0.1 mL, 1.58 mmol) was added and the reaction mixture was stirred for 24 h at rt. The solvent was evaporated and the obtained white powder was dissolved in methanol and subjected to DOWEX column (AcO^−^ form, exchanged from Cl^−^ via the OH^-^ form) to obtain the product with acetate counter ion as a yellow syrup (60 mg, 55%). ^1^H NMR (DMSO-d_6_): δ 1.87 (s, 3H, Me), 1.88 (s, Ac), 2.91 (s, 3H, MeN^+^), 7.35 (ddd, J_H-ortho,H-meta_ 7.63 and 0.37 Hz, J_H-ortho,H-para_ 1.17 Hz, 2H, H-ortho), 7.46 (dd, J_H-ortho,H-para_ 1.17 Hz, J_H-meta,H-para_ 7.52 Hz, 1H, H-para), 7.50 (ddd, J_H-ortho,H-meta_ 7.63 and 0.37 Hz, J_H-meta,H-para_ 7.52 Hz, 2H, H-meta) (Figure S11). ^13^C NMR (DMSO-d_6_): δ 12.13 (Me), 21.89 (AcO^-^), 32.14 (MeN^+^), 105.67 (C5), 128.77 (C-meta), 128.87 (C-para), 129.90 (C-ortho), 130.29 (C-ipso), 134.31 (C4), 161.91 (C2), 172.53 (CO) (Figure S12).

#### 4.1.4. Methyl l-glycero-α-d-manno-heptopyranosyl-(1→3)-l-glycero-α-d-manno-heptopyranoside (**C2**)

Reported in Supporting Information (Scheme S1, Figures S13 and S14).

### 4.2. Molecular Docking

Docking studies were performed by using four docking programs: AutoDock Vina [30], LeDock (http://lephar.com) [31], rDock [32] and GOLD [33], selected for their established sampling and scoring functions [34].

The crystal structures of WaaG, *viz.*, co-crystallized with uridine-5′-diphosphate-2-deoxy-2-fluoro-α-d-glucose (U2F), were retrieved by the Protein Data Bank (rcsb.org, PDB ID 2iw1) [46]. Four types of docking calculations were conducted with the molecular structures of libraries A, B and C. First, U2F was removed in order to analyze the docking-pose of ligands in a free binding pocket. Moreover, additional docking studies were performed with the protein in the presence of UDP-Glc. The donor ligand UDP-Glc was obtained by modifying the crystal structure of U2F with Avogadro [47] version 1.1.1. All molecules were visualized in PyMOL 2.3.4 or VMD [48] version 1.9.3. The docking studies were conducted on a PC with an Intel^®^ Core™ i7-9705H CPU @ 2.60 GHz with 8 GB RAM running Ubuntu 16.04 as operating system, unless otherwise stated.

The first employed docking program was AutoDock Vina 1.1.2. AutoDockTools (ADT) [49] version 1.5.6 was utilized to obtain not only the pdbqt files of both protein structures and all ligands but also to determine the different docking grid boxes (see Table S1). Docking was performed using the protein in its rigid form or by making some amino acids flexible, viz., Arg173, Arg261 and Glu289. These amino acids are known to establish important H-bonds with the uridine portion of UDP in the binding pocket of WaaG. The exhaustiveness value was set to 64 and the number of poses to 10 for each ADT run. The binding energy range was imposed to be ≤ 2 kcal·mol^−1^ above that of the best binding pose for each ligand.

LeDock was used as the second docking program. A file with all ligands in the mol2 (Tripos MOL2) format was generated from the original pdb files using Open Babel 2.3.2 [50]. LePro was employed for the pre-processing of the protein structure and to initiate the docking. Ten binding poses were generated from each docking run.

The third docking program employed was rDock. Input files in mol2 format of the protein and sdf (MDL SD) files of the ligands were utilized to carry out the docking. Using *rbcavity* to prepare the docking site, the two-sphere method was selected, and the radius of the sphere was set to 10 Å. The number of docking poses was set to 10 and the MDL SD output files reported the affinity scores for the generated poses.

GOLD was used as the last docking program. Pdb files of both protein structures and ligands were employed to execute GOLD docking predictions. The protein structure was properly protonated, and co-crystallized ligands and water molecules were removed. XYZ coordinates were edited and the binding site radius was set to 10 Å. Ligands were docked into the rigid form of the protein crystal structures, but also with some flexible side-chains, selected from the most important amino acids in the binding pocket. The amino acids were selected by checking how UDP-Glc interact with the protein and chosen in order to facilitate flexibility at the surface of the binding pocket. Ten amino acids were selected to be more flexible: Phe13, Asp100, Arg173, Arg208, Lys209, Arg261, Glu281, Ile285, Val286 and Glu289. By default, the number of dockings to be performed on each ligand was 10. Search efficiency (predictive reliability) was set to 100% (around 30.000 GA operations for each ligand). The docking studies were conducted on a PC with an Intel^®^ Core™ i7-9700H CPU @ 3.00 GHz with 16 GB RAM on Windows 10 Pro as the operating system.

### 4.3. Molecular Dynamics Simulations

MD simulations were performed with NAMD v. 2.12 [51]. Both protein and ligands files were generated employing the VMD psfgen tool. The CHARMM36 force field [52,53,54] was utilized, and the following sections of the CHARMM-GUI website were used: *PDB R&M* (Reader and Modeler) [55,56] for protein structures, *Ligand R&M* [57] for the molecules of libraries A and B; *Glycan R&M* [58,59,60] was used for the oligosaccharides of library C. The protein was protonated to a physiological pH of 7.2, with an overall net charge of +1.0 e. Ligands and ligand-protein complexes were solvated in a cubic water box, 50 Å to the side, using a TIP3P water model [61]. NaCl (0.2 M) ions were subsequently added and the system neutralized with the same ions.

An initial potential energy minimization (1000 steps) was conducted on solvent molecules and ions of the systems using conjugate gradient and line search algorithms, imposing a harmonic restraining potential to ligands and complexes of 500 kcal·mol^−1^·Å^−2^. A subsequent minimization was performed on solvated ligands or complexes without any restraints. A gradual heating step from 1 K to 310 K in 6 ps was applied to each system, which was then equilibrated during 600 ps. Production simulations of 10 ns were run with a timestep of 2, 1, or 0.1 fs in NPT (isothermal–isobaric) ensemble with a Langevin damping coefficient set to 1 ps^−1^. The temperature was kept constant at 310 K with a stochastic Langevin thermostat (damping coefficient of 1 ps^−1^), while the pressure was kept stable at 1 atm (an oscillating time of 100 fs and a damping time constant of 50 fs). The coordinates were recorded every 1 fs. Hydrogens of the water molecules were kept rigid using the ShakeH algorithm, and the PME (Particle Mesh Ewald) method was employed for the calculation of non-bonded interactions, with a 1 Å grid spacing. Electrostatic and van der Waals interactions were forced to zero at the cutoff distance of 12 Å (switching function from 10 Å). For ligand **B33**, a constraint of 1.64 Å between the chlorine atom and the lone pair (LP, as generated by CHARMM *Ligand Reader & Modeler*) was imposed, and it became important to keep the contact between the halogen and the LP stable during the entire MD simulations involving the *apo*-form of WaaG protein. MD simulations were performed at the National Supercomputer Centre (NSC), being part of Linköping University and the Swedish National Infrastructure for Computing (SNIC). Computations were carried out on the Tetralith cluster using Slurm to submit jobs and ThinLinc (Cendio AB, Linköping, Sweden) as a remote desktop.

### 4.4. Binding Free Energy Calculation

For post-MD analysis of free binding energy, the program CaFE (Calculation of Free Energy) 1.0 [62] was used to process PSF (coordinate) and DCD (trajectory) files generated by the CHARMM/NAMD (v. 2.12) software. CaFE was employed to predict binding affinity with an end-point method, viz., LIE (Linear Interaction Energy) [37,38], which is an approximation of the linear response [37]. PSF and DCD files of both the solvated molecule and in complex with the protein are required. CaFE generates for both settings a difference in electrostatic (polar term) and van der Waals (vdW) interactions (nonpolar terms) between the ligand and the enclosing background; then, the summation of these individual energy components gives the total binding free energy [62], reported as Δ*G*_calc_ and expressed in kcal·mol^−1^. The dissociation constant *K*_D_ was obtained from the free energy using the following relationship:

Δ*G* = *RT*ln(*K*_D_/c*)

where *R* is the ideal gas constant with a numerical value of 1.987×10^−3^ kcal⋅K^−1^⋅mol^−1^, *T* is the absolute temperature with a value of 310 K and c* is a standard reference concentration defined as 1 M [63]. CaFE employed the entire 10-ns MD trajectories of the solvated ligand and the complex. The CaFE script implemented the required CHARMM parameter files, and the entire MD trajectory was analyzed every 5^th^ frame by setting the stride to 5. The predefined coefficients in the LIE approach, *α*, β and *γ*, are taken as their default values, 0.18, 0.33 and 0.0, respectively.

### 4.5. RMSD Analysis

The root-mean-square-deviations (RMSDs) throughout the MD simulation were calculated during the 10-ns production period with the *RMSD Trajectory Tool* of VMD or each selected ligand. The reference structure is the first frame of the MD simulation. The RMSD analysis starts with a rigid-body structure alignment. Protein flexibility during the MD simulation was calculated in VMD. The stride was set to 1; therefore, each frame of the calculation was used for the RMSD analysis. RMSD values were then recorded and saved as a list of RMSDs at different frames, as a .dat file. Averaged RMSDs were calculated for each ligand, as well as the RMSD standard deviation (SD) values for each RMSD distribution. RMSD analysis was performed with MATLAB^®^ version 7.11.0.584 (R2020b), using in-house-built scripts.

### 4.6. MD Post-Processing: Interactions, Angle, Distance and Spearman Correlation

The electrostatic and van der Waals contributions on the binding between WaaG and each ligand were calculated after protein backbone alignment, through an in-house tcl script utilized in VMD Tk Console, which uses NAMD Energy 1.4 as its processing program. As for the H-bonds identification, after protein backbone alignment, the DCD and PSF files of each WaaG/ligand complex are imported in VMD; then, the unique hydrogen bonds are calculated, with a donor-acceptor distance tolerance of 3.4 Å and an angle cutoff of 45°. The angle and the distance variation between strategic amino acids were calculated through VMD, and the .dat output files were used for the Spearman correlation through an in-house script in MATLAB.

### 4.7. Saturation Transfer Difference NMR Spectroscopy

NMR spectroscopy experiments were carried out at 5 °C on a Bruker AVANCE 500 MHz spectrometer equipped with a 5 mm PFG triple-resonance CryoProbe (^1^H/^13^C/^15^N) and a Bruker AVANCE III 700 MHz equipped with a 5 mm TCI Z-Gradient Cryoprobe (^1^H/^13^C/^15^N). 1D STD NMR experiments were run using a protein concentration of ~25 μM, a ligand/protein ratio of 20:1 and 600 µM UDP concentration when used. NMR tubes with an outer diameter of 3 mm (0.18 mL) were utilized for the NMR analysis. On-resonance irradiation for protein saturation was set at 0.5, 0 and −0.5 ppm, while the off-resonance control was positioned at 60 ppm using Gaussian pulses at a power level corresponding to a hard square pulse of 65 Hz and a length of 50 ms. An acquisition time of 2 s and a relaxation delay of 6 s was employed for a total number of 21,928 data points. An excitation sculpting for water suppression was employed with a squa100.1000 function of 2400 μs, while the TOCSY spinlock was set to 50 ms at 5 kHz. A total number of scans of 1024 was employed for each experiment. Blank control 1D STD experiments were run in the same way, with the sample containing only the ligand without the presence of the protein.

### 4.8. Sample Preparation for STD NMR Spectroscopy

Protein preparations in pure glycerol medium were kept at −80 °C and were thawed slowly to attain 0 °C when used. Three cycles of buffer exchange using Amicon Ultra centrifuge were run for 15 min each at 14,000× *g* and 4 °C. Concentration of the protein samples was obtained using a BioSpec-Nano spectrophotometer via optical density measurement at a wavelength of 280 nm, employing a 42,284 g·mol^−1^ reference value for WaaG. The samples were prepared in a phosphate-buffered saline (PBS) solution containing KH_2_PO_4_ (25 mM) and NaCl (150 mM) in D_2_O and the pD adjusted to 8.6 with DCl and NaOD.

### 4.9. Biochemical In Vitro Assessment/In Vitro WaaG Activity Assay

The WaaG activity assay was performed as previously described [23], using herein ligands of libraries A and B.

## 5. Conclusions

Conformational aspects and the presence of dynamics in WaaG were investigated by considering the entire protein backbone, or the single parts, i.e., the N- and C-domains and the hinge region. A dynamic behavior of the two domains was observed, with movement relative to each other in a twisting-type fashion. Restricting the movements or interfering with the inherent dynamics of WaaG by using small molecule inhibitors that can act by ‘putting a spanner in the works’ could open a way to potentiate the action of antibiotics in pathogenic *E. coli* and other gram-negative bacteria.

## Figures and Tables

**Table 1:** Computed Δ*G*_bind_ (kcal·mol^−1^) and calculated *K*_D_ values for ligands of libraries A, B and C. Errors in Δ*G*_bind_ are estimated to be 1–2 kcal·mol^−1^, corresponding to one order of magnitude in calculated *K*_D_ values.

LIGAND	Δ*G*_bind_	Calc *K*_D_ (mM)	LIGAND	Δ*G*_bind_	Calc *K*_D_ (mM)	LIGAND	Δ*G*_bind_	Calc *K*_D_ (mM)
**A7**	−5.65	0.11	**B33**	−6.29	0.04	**C4^2−^**	−9.14	0.0004
**A4**	−4.58	0.62	**B16**	−3.89	1.89	**C4**	−8.70	0.0008
**A9**	−3.34	4.55	**B22**	−3.18	5.97	**C2**	−5.97	0.07
**A15**	−1.73	61.10	**B3d**	−2.19	29.09	**C3**	−4.21	1.13
**A14**	−0.81	269.26	**B26**	−1.03	190.48	**C1**	−4.20	1.15
**A1**	−0.64	354.70				**C3^1−^**	−0.72	313.08
**A8**	>0	> 10^3^						
**A16**	>0	> 10^3^						

## Data Availability

Data is contained within the article.

## Supplementary Materials

The following are available online at https://www.mdpi.com/article/10.3390/ph15020209/s1, Figure S1: Ligands of library A, Figure S2: Ligands of library B; Figure S3: Ligands of library B; Figure S4: Ligands of library C; Figure S5: Ligands of library C; Table S1: Parameters for dockings of ligands to WaaG; Table S2: Ranking of ligands in docking to WaaG; Table S3: Average RMSD on WaaG backbone during MD simulations on WaaG/ligand complexes; Figure S6: RMSDs calculated for the protein backbone during MD simulations; Figure S7: 1H NMR spectrum of compound A11; Figure S8: 13C NMR spectrum of compound A11; Figure S9: 1H NMR spectrum of compound A12; Figure S10: 13C NMR spectrum of compound A12; Figure S11: 1H NMR spectrum of compound A13; Figure S12: 13C NMR spectrum of compound A13; Scheme S1: Synthesis of heptobioside C2; Figure S13: 1H NMR spectrum of compound C2; Figure S14: 13C NMR spectrum of compound C2; ligand-protein complexes in pdb format (9 files).
