# Supplementary material for: A Lead-Based Fragment Library Screening of the Glycosyltransferase WaaG from Escherichia coli
Source: Pharmaceuticals (Basel). 2022 Feb 9;15(2):209. doi: 10.3390/ph15020209 (PMC8877264; doi:10.3390/ph15020209)
Supplement: Supplementary file 1 [file pharmaceuticals-15-00209-s001.zip › pharmaceuticals-1578293/pharmaceuticals-1578293 (5).pdf]

A Lead-Based Fragment Library Screening of the Glycosyltransferase WaaG from *Escherichia coli*

Federico Riu[a], Alessandro Ruda[b], Olof Engström[b], Claudio Muheim[c], Hani Mobarak[b], Jonas Ståhle[b], Paul Kosma[d], Antonio Carta[a], Daniel O. Daley[c], Göran Widmalm[b]

[a] Department of Medical, Surgical and Experimental Sciences, University of Sassari, Via Muroni, 23A, 07100 Sassari, Italy

[b] Department of Organic Chemistry, Arrhenius Laboratory, Stockholm University, S-106 91 Stockholm, Sweden

[c] Department of Biochemistry and Biophysics, Arrhenius Laboratory, Stockholm University, S-106 91 Stockholm, Sweden

[d] Department of Chemistry, University of Natural Resources and Life Sciences-Vienna, 1190 Vienna, Austria

Supporting Information

Table of Contents

Table of Contents..........S1

Compounds of libraries A, B and C..........S2 – S5

Molecular Docking parameters and ranking..........S6

Molecular Docking of ligands to the WaaG-UDP-Glc^{2-} complex..........S7 – S8

MD-related average RMSDs and related standard deviations for WaaG/ligand complexes..........S8

MD-related RMSDs for the *apo*-protein and the WaaG-ligand complexes..........S9

^{1}H and ^{13}C NMR spectra of synthesized compounds **A11**, **A12** and **A13**..........S10 – S12

Synthetic route for heptobioside **C2**..........S13

General methods and procedures of intermediates and **C2**..........S14 – S16

^{1}H and ^{13}C NMR spectra of **C2**..........S17 – S18

References..........S18

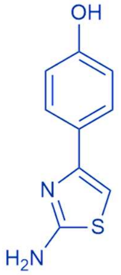

A1

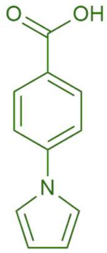

A2

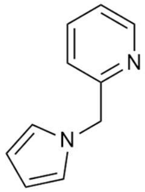

A3

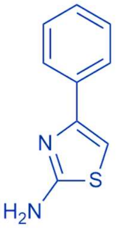

A4

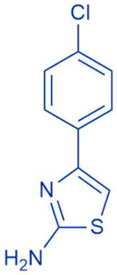

A5

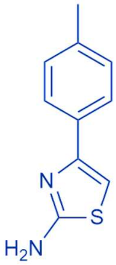

A6

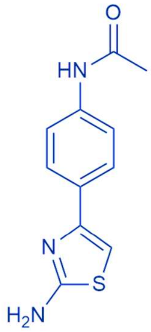

A7

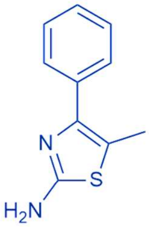

A8

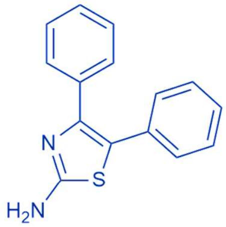

A9

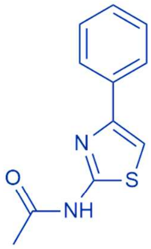

A10

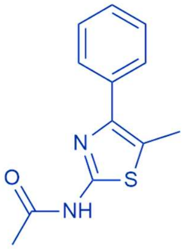

A11

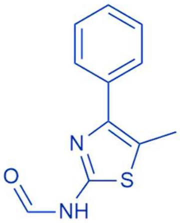

A12

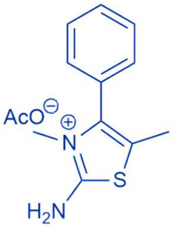

A13

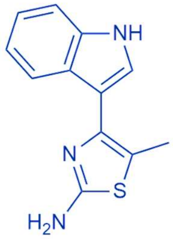

A14

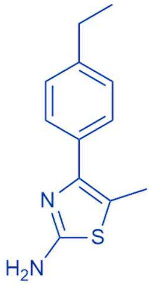

A15

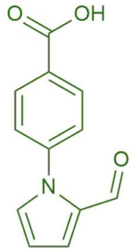

A16

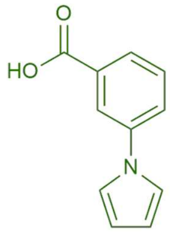

A17

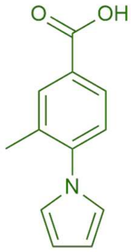

A18

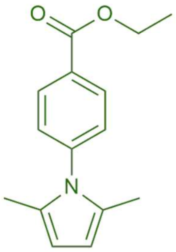

A19

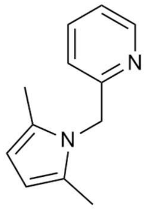

A20

Figure S1. Ligands of library A (**A1** – **A20**). **A1**-derived compounds **A4** – **A15** are coloured in blue, **A2**-derived compounds **A16** – **A19** are coloured in green and **A3**-derived compound **A20** is in black.

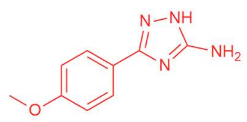

B1

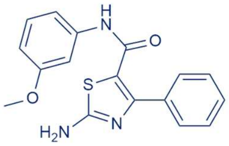

B2

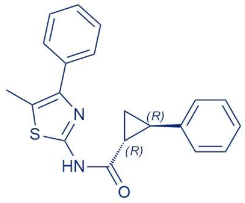

B3a

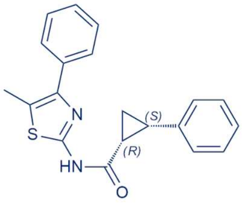

B3b

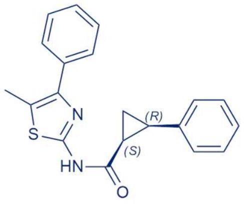

B3c

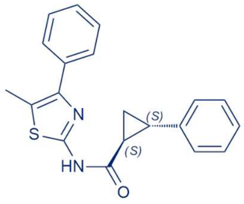

B3d

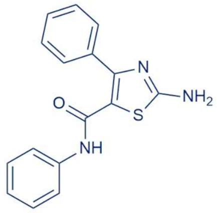

B4

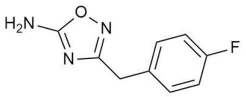

B5

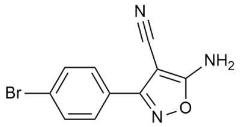

B6

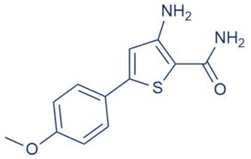

B7

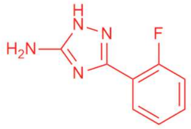

B8

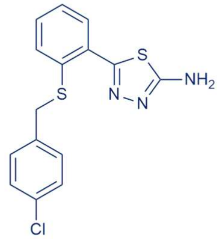

B9

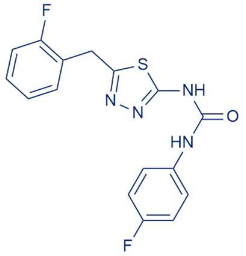

B10

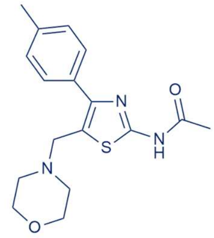

B11

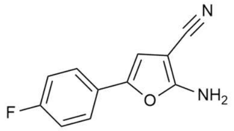

B12

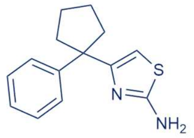

B13

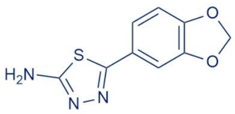

B14

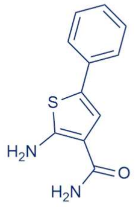

B15

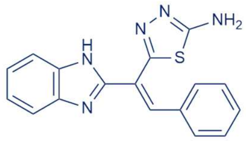

B16

Figure S2. Ligands of library B (**B1** − **B16**). Triazole-derived compounds **B1** and **B8** are coloured in red. Thiazole-derived compounds **B2** – **B4**, **B7**, **B9** − **B11**, **B13** − **B16** are coloured in blue. The other compounds (**B5**, **B6**, **B12**) are coloured in black.

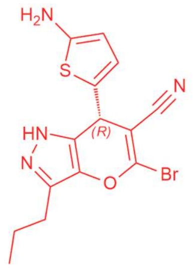

B17a

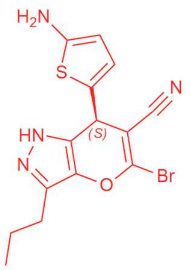

B17b

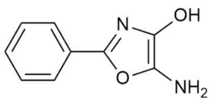

B18

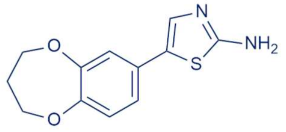

B19

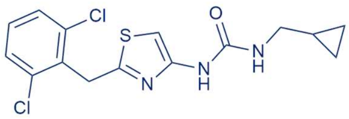

B20

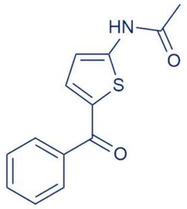

B21

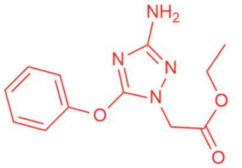

B22

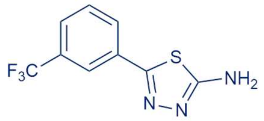

B23

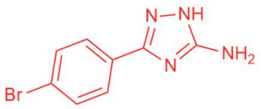

B24

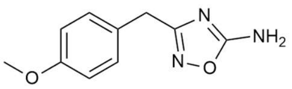

B25

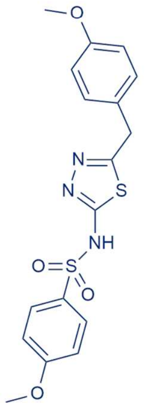

B26

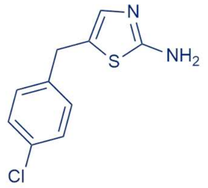

B27

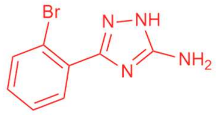

B28

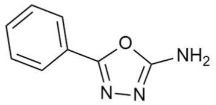

B29

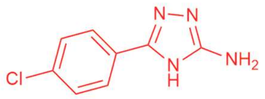

B30

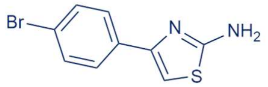

B31

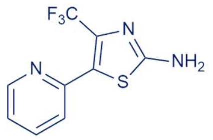

B32

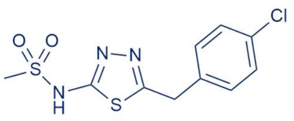

B33

Figure S3. Ligands of library B (**B17a** − **B33**). Pyrazole- or triazole-derived compounds **B17a** − **B17b, B22, B24, B28** and **B30** are coloured in red. Thiophene, thiadiazole or thiazole-derived compounds **B19** − **B21**, **B23**, **B26** − **B27**, **B31** − **B33** are coloured in blue. The other compounds (**B18**, **B25**, **B29**) are coloured in black.

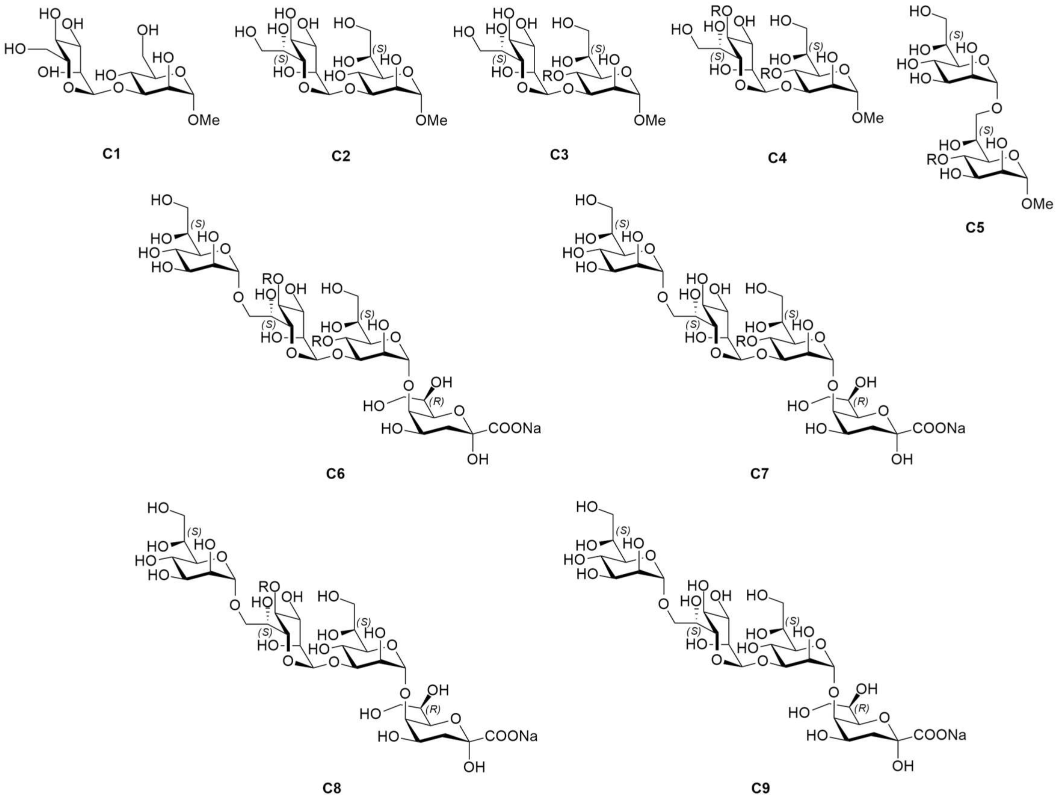

Figure S4. Ligands of library C (**C1** − **C9**). R = HPO_3Na.

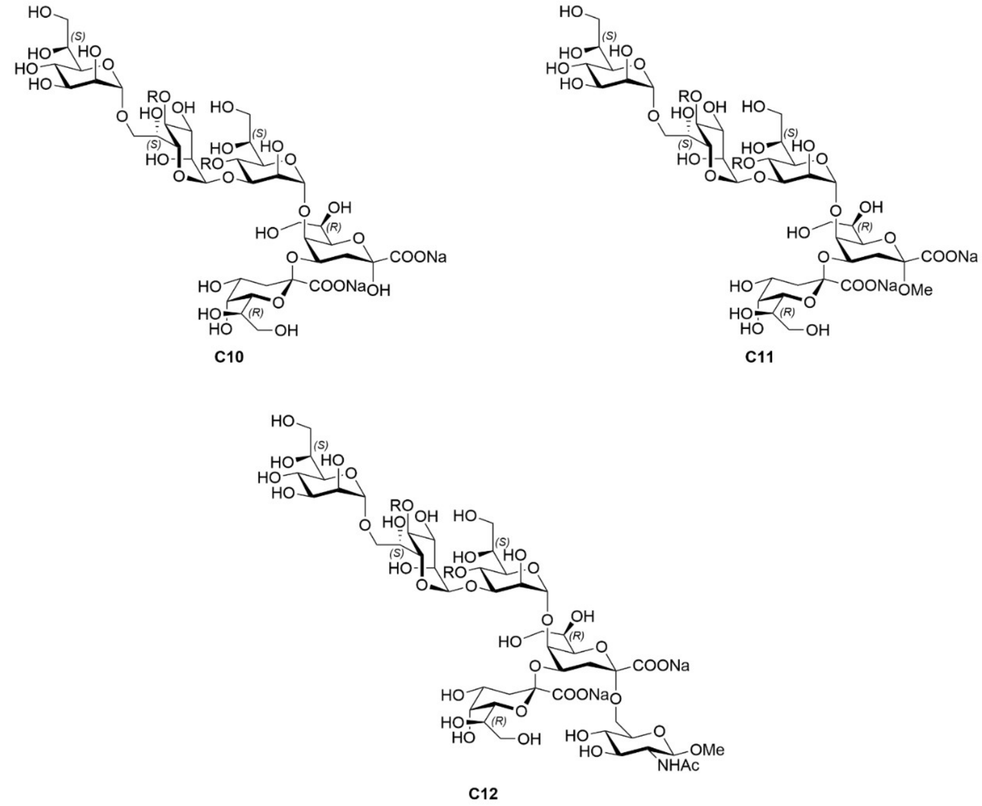

Figure S5. Ligands of library C (**C10** − **C12**). R = HPO_3Na.

Table S1. Parameters for dockings of ligands to WaaG (Libraries A/B/C).

***apo* -WaaG**			
**Docking program**	**Active site center** (x,y,z)	**Active site dimension** (x,y,z)	**Flexible aminoacids**
AutoDock Vina	-5.694/10.389/-17.222	22-22-22 points	
	-5.639/10.528/-19.833	24-26-26 points	
	-5.694/10.389/-17.222	22-22-22 points	Arg173-Arg261-Glu289
LeDock	-5.694/10.389/-17.222	22-22-22 points	
rDock	-5.694/10.389/-17.222	10.0 radius	
GOLD	-5.694/10.389/-17.222	16 Å	
	-5.694/10.389/-17.222	16 Å	Phe13-Asp100-Arg173-Arg208-Lys209-Arg261-Glu281-Ile285-Val286-Glu289

**WaaG+UDPGlc**			
**Docking program**	**Active site center** (x,y,z)	**Active site dimension** (x,y,z)	**Flexible aminoacids**
AutoDock Vina	-3.000/22.306/-17.222	18-22-22 points	
LeDock	-2.083/24.750/-16.583	18-22-22 points	
rDock	-3.000/22.306/-17.222	10 Å radius	
GOLD	-3.000/22.306/-17.222	18 Å radius	

Table S2. Ranking of ligands in docking to WaaG (Libraries A/B/C).

**Ranking of ligands in docking to WaaG**																	
		***apo* -WaaG**[a]								**WaaG+UDPGlc**[a]							
	#	AD	Affinity E	LD	Score	rD	Score	G	Score	AD	Affinity E	LD	Score	rD	Score	G	Score
**A**	1	**9**	-8.7	**7**	-7.2	**1**	-34.6	**16**	62.6	**9**	-7.8	**7**	-6.6	**1**	-42.8	**14**	53.9
	2	**11**	-8.0	**11**	-6.4	**7**	-33.8	**19**	60.0	**15**	-7.3	**9**	-6.3	**13**	-37.8	**1**	52.2
	3	**7**	-8.0	**14**	-6.3	**9**	-30.2	**18**	59.7	**14**	-7.2	**11**	-6.1	**17**	-37.5	**7**	51.0
	4	**14**	-7.9	**9**	-6.3	**14**	-30.0	**17**	58.1	**12**	-7.1	**10**	-6.0	**14**	-37.3	**15**	51.0
	5	**16**	-7.3	**10**	-6.2	**15**	-29.4	**11**	56.0	**16**	-7.0	**12**	-6.0	**8**	-36.9	**5**	50.4
**B**	1	**3d**	-9.8	**26**	-9.5	**22**	-39.1	**3d**	77.8	**16**	-8.8	**16**	-8.3	**33**	-42.1	**16**	64.7
	2	**10**	-9.7	**10**	-8.9	**16**	-38.0	**3a**	77.7	**3d**	-9.1	**3d**	-6.6	**18**	-38.3	**3d**	64.5
	3	**3a**	-9.7	**33**	-8.8	**7**	-36.9	**10**	76.8	**3a**	-8.5	**3a**	-6.9	**13**	-38.2	**10**	63.2
	4	**2**	-9.3	**16**	-8.4	**17a**	-35.5	**26**	71.2	**10**	-8.8	**10**	-8.7	**28**	-37.8	**9**	62.1
	5	**26**	-8.8	**9**	-8.2	**6**	-33.3	**20**	70.7	**3b**	-8.5	**3b**	-6.1	**29**	-37.1	**26**	61.7
**C**	1	***6***	-8.8	***6***	-11.5	***6***	-180.0	***10***	79.9	***8***	-8.4	***10***	-15.1	***6***	-94.2	***10***	73.7
	2	***9***	-8.8	***8***	-11.2	***3***	-51.9	***9***	79.3	***10***	-7.6	***6***	-14.0	***5***	-22.3	***11***	67.2
	3	***8***	-8.7	***9***	-11.1	***4***	-51.6	***7***	76.1	***5***	-7.5	***8***	-13.8	***3***	-19.7	***12***	63.1
	4	***10***	-8.5	***4***	-10.0	***5***	-47.0	***6***	70.6	***7***	-7.5	***7***	-12.8	**2**	-19.4	***6***	55.9
	5	***5***	-8.2	***3***	-9.5	**1**	-34.7	***5***	66.3	***6***	-7.4	***9***	-12.0	**1**	-17.6	***7***	55.4

[a] Crystal structure used (PDB ID: 2iw1).

AD = AutoDock Vina; Affinity E = Affinity Energy; LD = LeDock; rD = rDock; G = GOLD.

Italics for phosphate-substituted oligosaccharides.

Molecular docking of ligands to the WaaG-UDP-Glc^{2-} complex

The donor UDP-Glc^{2-} docks in a narrow portion of WaaG-binding pocket, whereas the outermost space of the protein active site has a large cavity. A prominent sub-pocket is present in the outer portion of the binding site, represented by the amino acids Lys89, Tyr103, Ala104, Phe127 and Glu128 (outer sub-pocket, *abbr.* OSP). Docking was conducted in the presence of the WaaG-UDP-Glc^{2-} complex (2iw1) and each ligand of the different libraries.

From library A, ligand **A9** showed the best binding affinity for the AD Vina docking studies with the complex WaaG-UDP-Glc^{2-}, while ligands **A7** and **A14** had LeDock and GOLD top-ranked poses, respectively. Considering the affinity energy ranks of the different docking programs, ligands **A15** and **A1** were ranked with their top-predicted poses for the docking calculations involving the complex WaaG-UDP-Glc^{2-}. In library A, compounds **A1**, **A4**, **A7** and **A9** showed the best affinity results when docked with the WaaG-UDP-Glc^{2-} complex. The aminothiazole moiety has a key role in the binding of ligands from library A, like **A1**, **A7** and **A9**, binding in the inner portion of OSP region. The best-predicted pose for **A7** exposes the amide part towards the outer portion of the WaaG binding pocket. The only ligand of the selected ones to have the aminothiazole ring pointing towards the outer part of the pocket is **A4**.

In library B, compound **B16** has the best-predicted pose for docking programs AutoDock Vina, LeDock and GOLD, while the top-predicted pose of ligand **B33** ranked the best protein-ligand affinity for rDock calculations. The benzyl moiety of **B16** docks in the inner portion of the OSP region, while its benzimidazole NH contacts the carbonyl oxygen of Asn88. Ligand **B33** docks its *p*-chlorobenzene ring in the inner OSP region, while the remaining part of the molecule is located along the curved portion between the UDP-Glc^{2-} binding region and the OSP portion. The sulfonamide oxygen in **B33** plays a key role in the ligand-protein binding where it as an H-bond acceptor atom is in contact with NH of the side-chain amide group of Gln280

As for library C oligosaccharides, disaccharide **C1** does not bind in the above-mentioned OSP region, but it is located near the outer UDP-Glc^{2-} binding region, where the glucose residue resides. Similar to **C1**, ligand **C2** docks the reducing-end heptose residue closer to the glucose binding region, as well as the heptose-4*P* residue of compound **C3**. Both derivatives **C4** and **C5** point towards the glucose binding region; in particular, the phosphates are closer to this inner region. The phosphate group of Hep-II in the best-predicted pose of the tetrasaccharide **C6** interacts with glucose and β-phosphate residues of UDP-Glc^{2-}, exposing the Kdo side chain in the outer part of the binding pocket. The Hep-III residue in both compounds **C7** and **C8** docks at the interface of the glucose binding site while the rest of the molecule is located in the outer part of the binding pocket. Tetrasaccharide **C9** docks its

Hep-II residue as to be exposed to the glucose binding region, while the best-docked poses of **C10** and **C11** expose the Hep-III moiety to the β-phosphate and glucose of UDP-Glc^{2-} binding region. Compound **C12**, instead, docks its Kdo residue at the interface of the glucose binding region, and the *N*-acetyl group of GlcNAc in the OSP region.

Notably, disaccharide **C1** was docked with the hydroxyl group in position 3 of the non-reducing end mannose toward the anomeric carbon of glucose in UDP-Glc^{2-}; the distance between O3 in the mannosyl residue and C1 in UDP-Glc^{2-} was in this pose 3.5 Å. A similar spatial arrangement was observed for ligand **C2** with an oxygen-carbon distance of 3.4 Å between the corresponding atoms in the heptose residue and UDP-Glc^{2-}. However, ligands **C3** and **C5** were docked in the binding pocket of WaaG with Hep-I hydroxyl oxygen atoms on C6 and C7 pointing towards C1 of glucose in UDP-Glc^{2-}.

Table S3. Average RMSD (avRMSD) in Å and related standard deviations (SD) calculated on WaaG backbone during each of the 10-ns MD simulations on WaaG/ligand complexes.

	avRMSD	SD
**apo**	1.31	0.16
**A1**	1.97	0.28
**A4**	1.43	0.21
**A7**	1.63	0.22
**A8**	1.78	0.56
**A9**	1.98	0.27
**A14**	1.33	0.25
**A15**	1.56	0.29
**A16**	1.37	0.40
**B3d**	1.43	0.21
**B16**	1.63	0.22
**B22**	1.96	0.38
**B33**	1.29	0.25
**C1**	2.54	1.24
**C2**	2.04	0.46
**$C3^{1-}$**	1.29	0.30
**C3**	1.35	0.21
**$C4^{2-}$**	1.24	0.14
**C4**	2.03	0.50

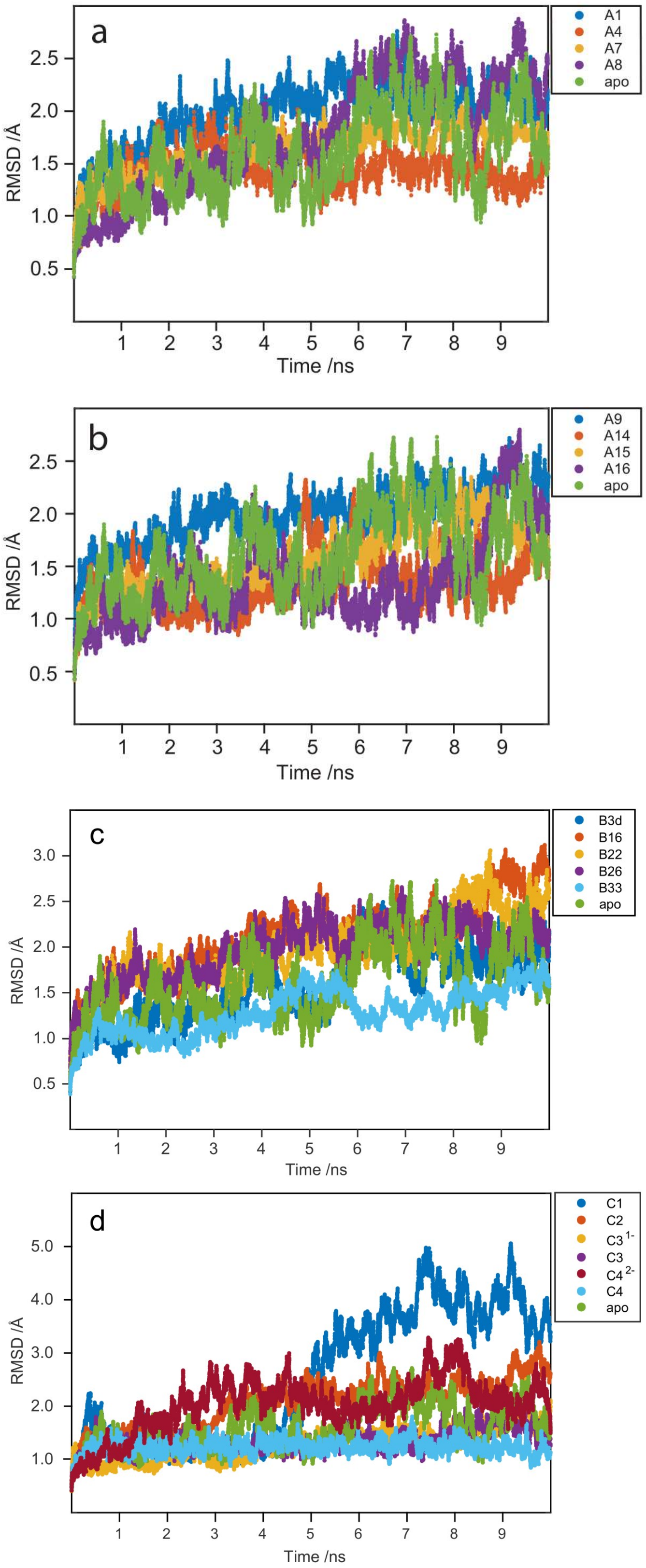

Figure S6. RMSDs calculated for the protein backbone of the *apo*-protein (“apo”, from 2iw1) and the various WaaG-ligand complexes of library A (a), B (b, c) and C (d) during the 10-ns of MD simulations (production run) on each WaaG-ligand complex.

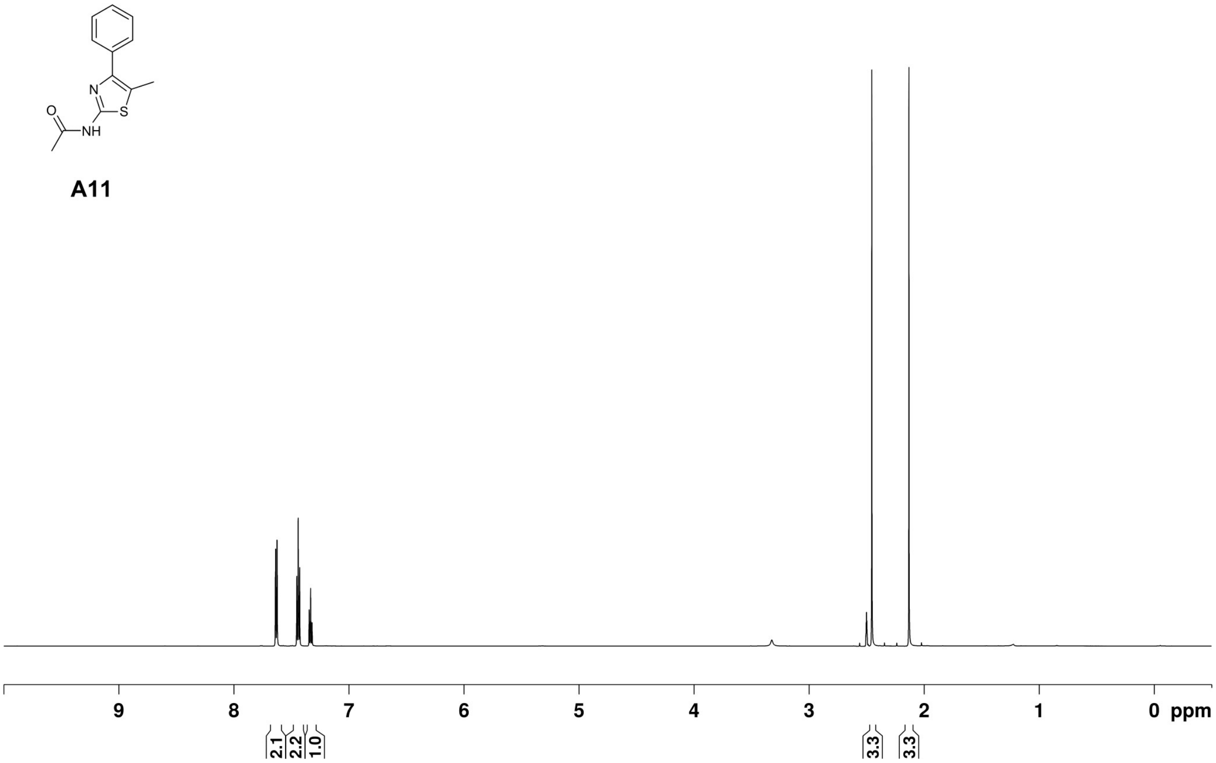

Figure S7. ^{1}H NMR spectrum of compound **A11**.

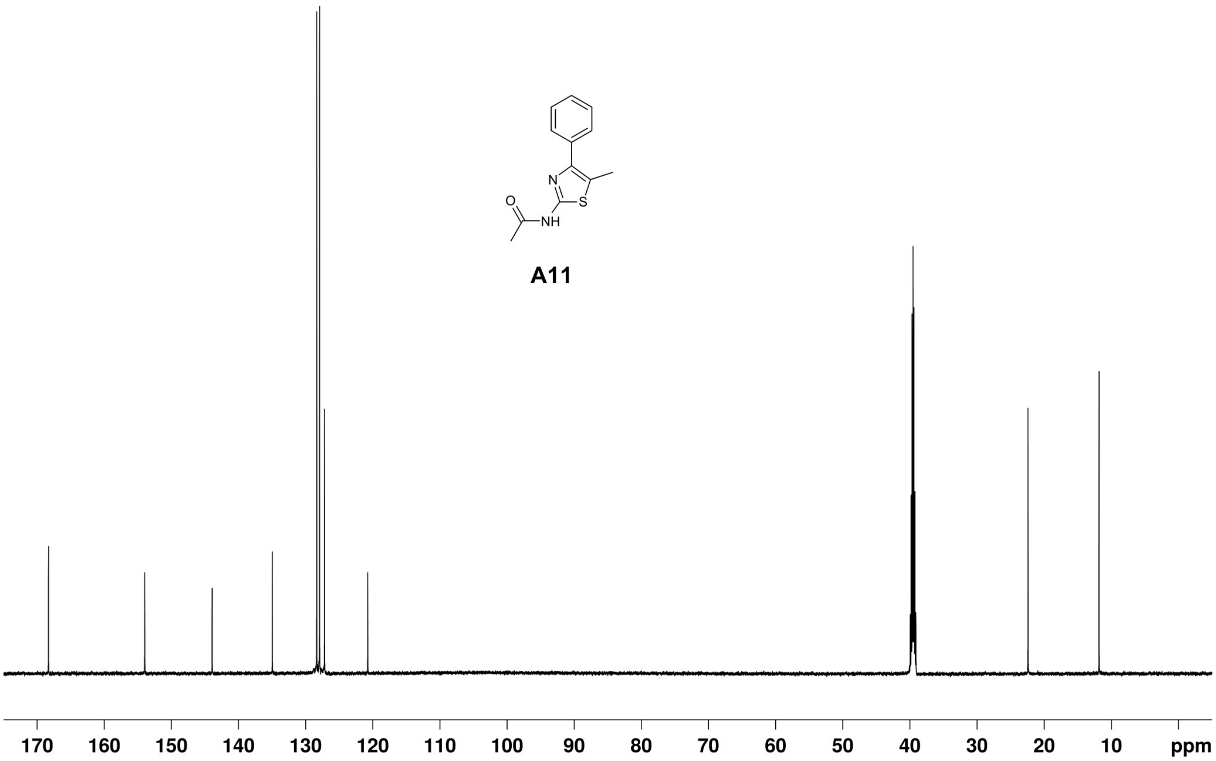

Figure S8. ^{13}C NMR spectrum of compound **A11**.

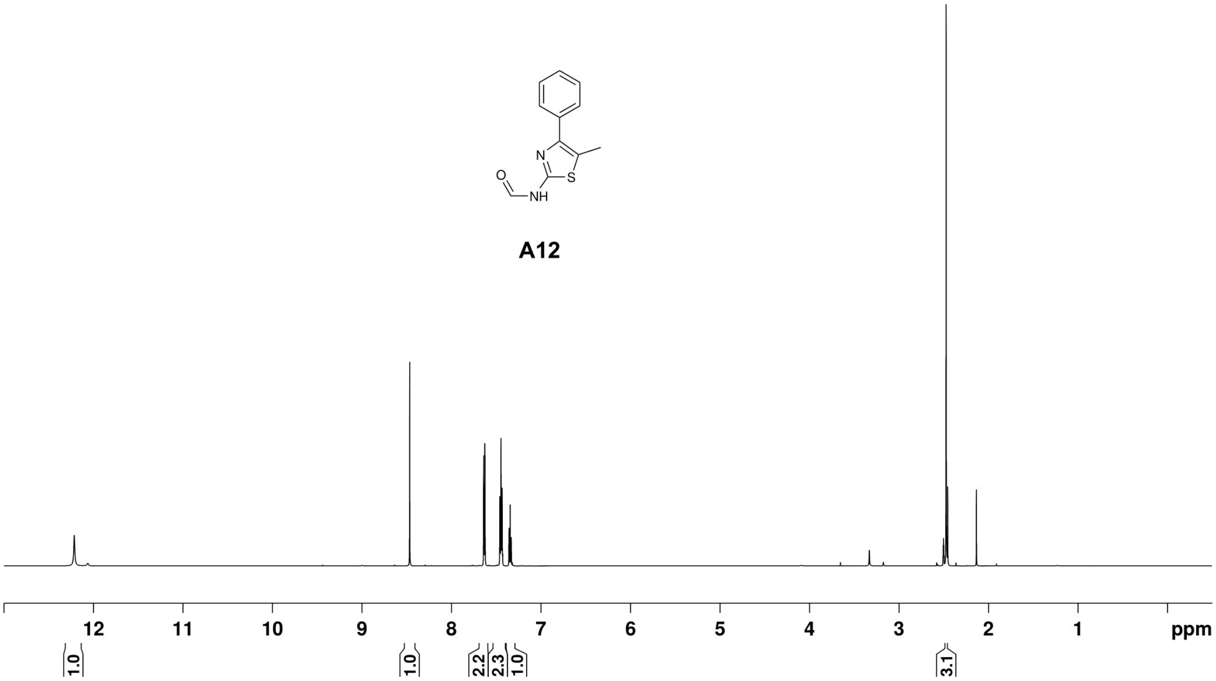

Figure S9. ^{1}H NMR spectrum of compound **A12**.

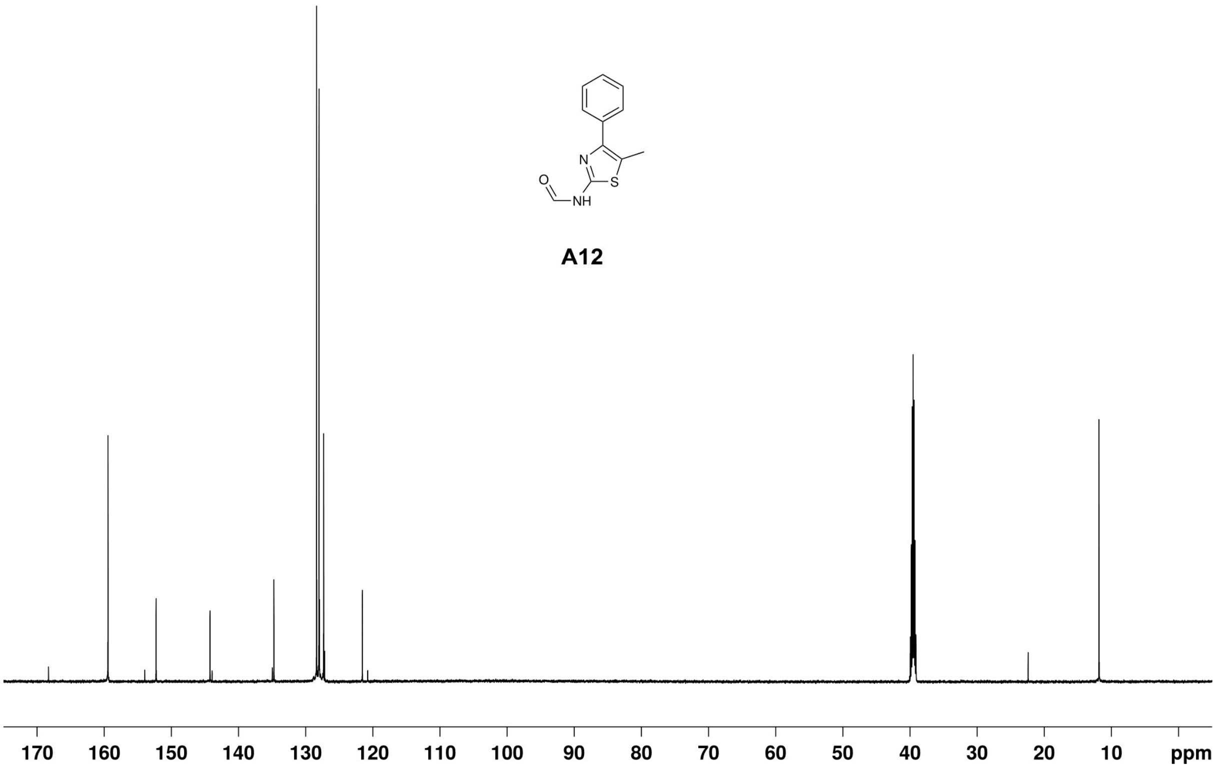

Figure S10. ^{13}C NMR spectrum of compound **A12**.

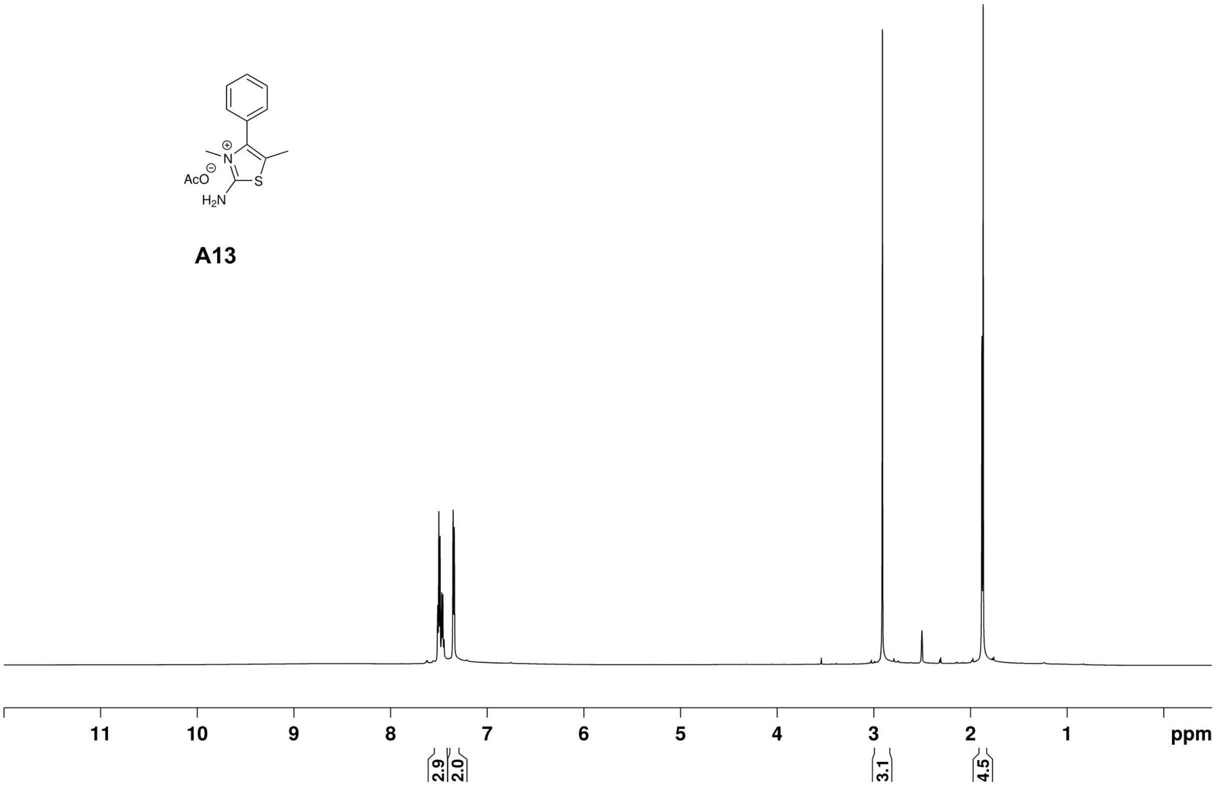

Figure S11. ^{1}H NMR spectrum of compound **A13**.

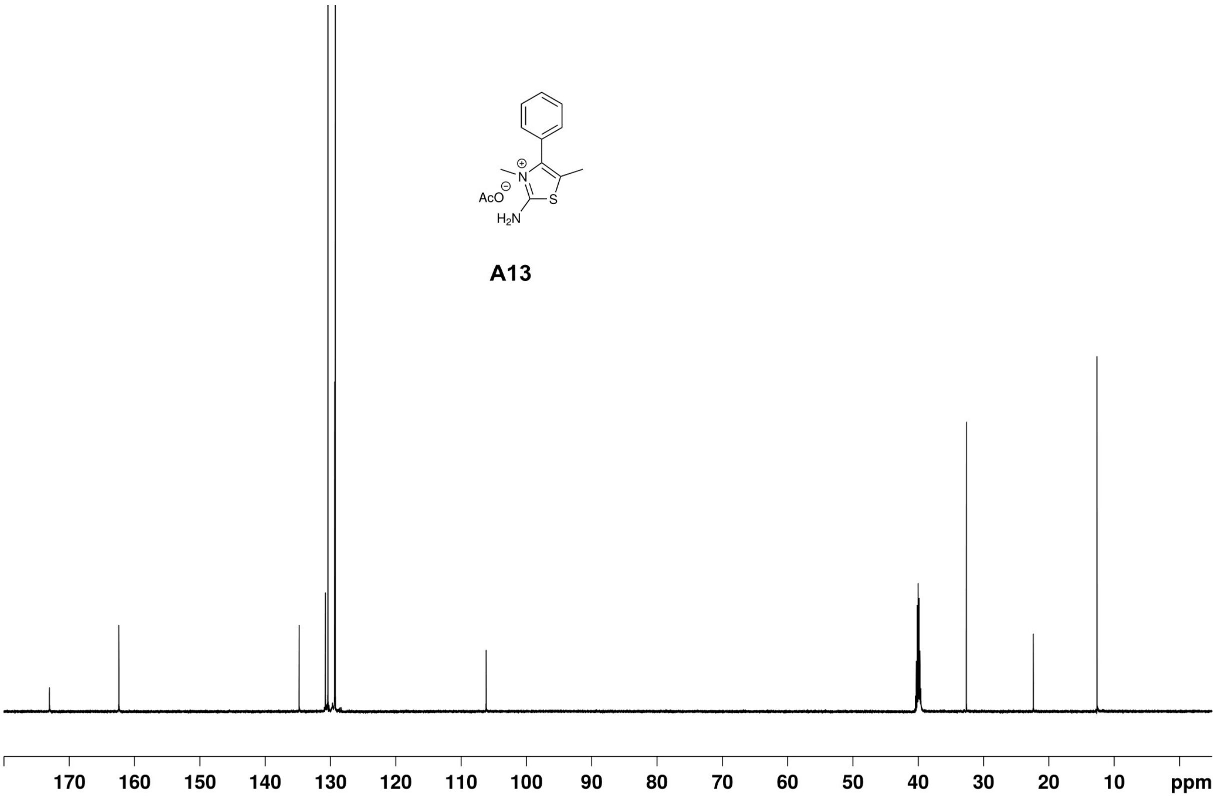

Figure S12. ^{13}C NMR spectrum of compound **A13**.

Synthesis of acceptor C14:

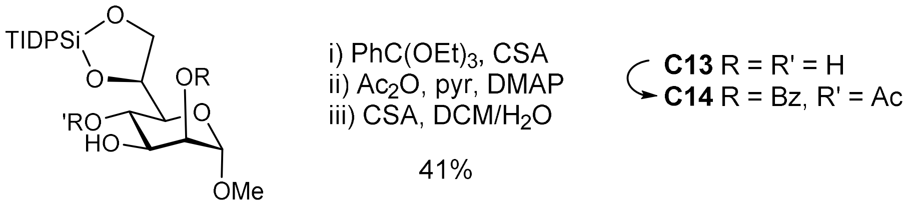

Synthesis of compound C2:

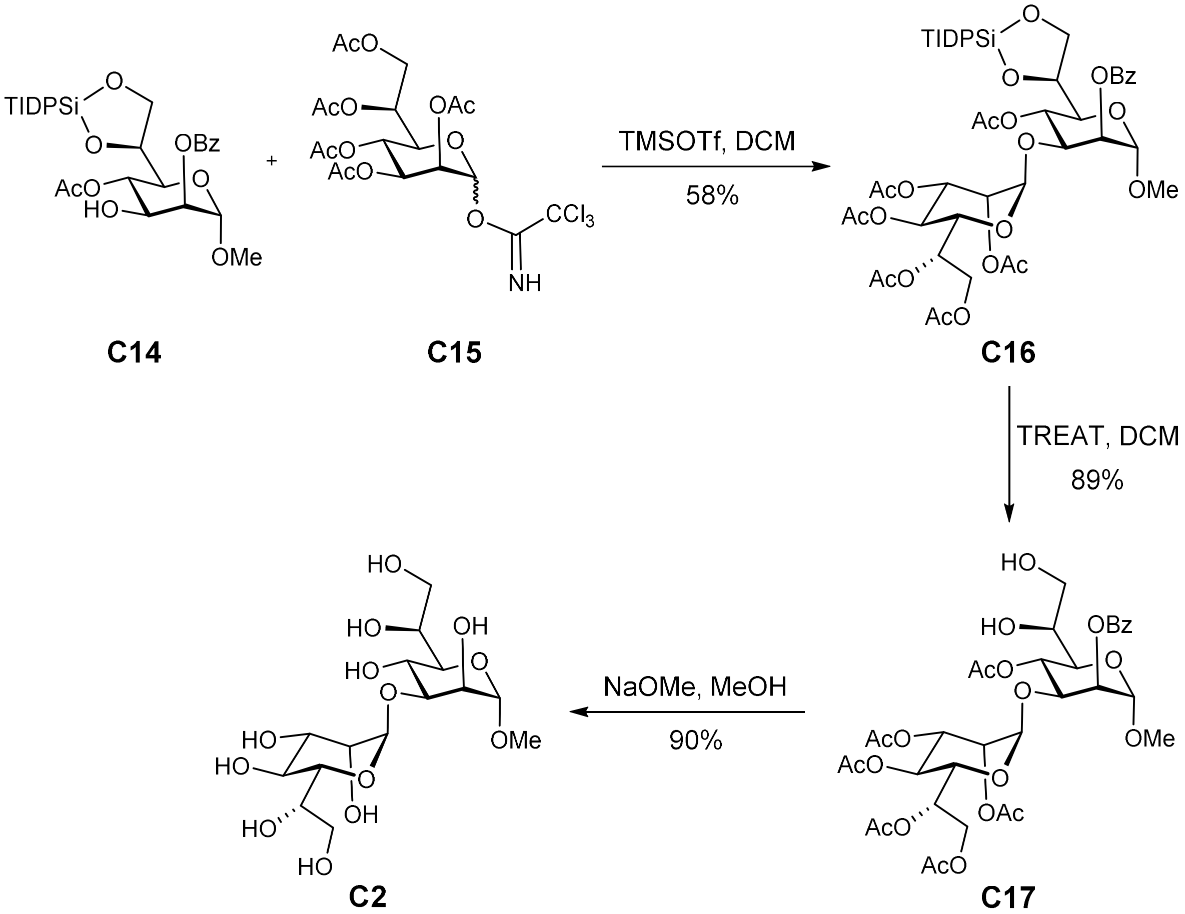

Scheme S1. Synthesis of heptobioside **C2**.

The disaccharide was prepared according to Scheme S1 by TMSO-triflate promoted coupling of trichloroacetimidate donor **C15** to glycosyl acceptor **C14**, followed by global deprotection.

General Methods

All purchased chemicals were used without further purification unless stated otherwise. CH_2Cl_2 was dried over activated 4 Å molecular sieves. Cation exchange resin DOWEX 50 H^+ was regenerated by consecutive washing with HCl (3 M), water and dry MeOH. Aqueous solutions of salts were saturated unless stated otherwise. Concentration of organic solutions was performed under reduced pressure <40 °C. Optical rotations were measured with an Anton Paar MCP100 Polarimeter. Thin-layer chromatography was performed on Merck precoated plates: generally, on 5 × 10 cm, layer thickness 0.25 mm, Silica Gel $60F_{254}$; alternatively, on HPTLC plates with 2.5 cm concentration zone (Merck). Spots were detected by staining with a dipping reagent (anisaldehyde-H_2SO_4) and heating. For column chromatography silica gel (0.040 – 0.063 mm) was used. HP-column chromatography was performed on pre-packed columns (YMC-Pack SIL-06, 0.005 mm, 25 × 1 cm and 25 × 2 cm). NMR spectra were recorded with a Bruker Avance III 600 instrument (600.22 MHz for ^1H, 150.93 MHz for ^{13}C) using standard Bruker NMR software. ^1H NMR spectra were referenced to TMS (δ_H 0.0) or externally to 2,2-dimethyl-2-silapentane-5-sulfonic acid for solutions in D_2O. ^{13}C NMR spectra were referenced to 77.00 ppm ($CDCl_3$) and 67.40 ppm (external calibration to 1,4-dioxane in D_2O). Resonance assignments were based on COSY, HSQC, and HMBC data. ESI-MS data were obtained on a Micromass Q-TOF Ultima Global instrument.

*Methyl 4-O-acetyl-2-O-benzoyl-6,7-O-(1,1,3,3-tetraisopropyl-1,3-disiloxane-1,3-diyl)-L-glycero-α-D-manno-heptopyranoside (***C14**). A catalytic amount of camphorsulfonic acid (2.5 mg) was added to a solution of **C13**[1] (100 mg, 0.214 mmol) and triethylorthobenzoate (58.2 µL, 0.257 mmol) in DCM (3 mL) at rt. The solution was stirred for 30 min followed by addition of triethylamine (30 µL). The solution was concentrated and coevaporated with toluene. The residue was dissolved in dry pyridine (2 mL) and acetic anhydride (81 µL, 0.857 mmol) and DMAP (1.3 mg) were added. The solution was stirred for 5 h at rt and cooled to ice-bath temperature. MeOH (173 µL) was added and stirring was continued for 30 min. The solution was concentrated and coevaporated with toluene. The residue was dissolved in DCM (3 mL) and treated with water (11 µL) and CSA (2.5 mg) for 3 h at rt. The solution was diluted with EtOAc and washed with satd aq. $NaHCO_3$. The aqueous phase was again extracted with EtOAc and the combined organic layer was dried (Na_2SO_4) and concentrated. The residue was purified by silica gel chromatography (hexane-EtOAc 20:1→5:1) to afford **C14** as colourless syrup. Yield: 53.4 mg (41%). ^1H NMR (600 MHz, $CDCl_3$): δ 8.08 (dd, 2 H, J 7.8, 1.4 Hz, arom. H), 7.59 (dt, 1 H, J 7.5, 1.4 Hz, arom. H), 7.44 (br t, 2 H, J 7.8 Hz, arom. H), 5.46 (t, 1 H, $J_{2,3} = J_{4,3}$ 9.9 Hz, H-4), 5.29 (dd, 1 H, $J_{2,3}$ 3.4, $J_{2,1}$ 1.7 Hz, H-2), 4.84 (d, 1 H, $J_{1,2}$ 1.5 Hz, H-1), 4.29 (dt, 1 H, $J_{6,5}$ = $J_{6,7b}$ 1.4, $J_{6,7a}$ 8.7 Hz, H-6), 4.10 (dd, 1 H, $J_{2,3}$ 3.5, $J_{4,3}$ 9.2 Hz, H-3), 4.09 (dd, 1 H, $J_{7a,7b}$ 12.3 Hz,

H-7a), 3.90 (dd, 1 H, H-7b), 3.80 (dd, 1 H, H-5), 3.37 (s, 3 H, CH_3O), 2.47 (d, 1 H, *J* 8.8 Hz, OH), 2.13 (s, 3 H, CH_3CO), 1.30 [m, 4 H, 4 × C*H*(CH_3)$_2$] and 1.13-0.95 [m, 24 H, 4 × SiCH(C*H*$_3$)$_2$].

Methyl (2,3,4,6,7-penta-O-acetyl-L-glycero-α-D-manno-heptopyranosyl)-(1→3)-4-O-acetyl-2-O-benzoyl-6,7-O-(1,1,3,3-tetraisopropyl-1,3-disiloxane-1,3-diyl)-L-glycero-α-D-manno-heptopyranoside (**C16**). A suspension of **C14** (50.0 mg, 0.082 mmol) and donor **C15** (72.3 mg, 0.128 mmol) and powdered molecular sieves 4 Å (70 mg) was stirred for 45 min at rt. Then 4 portions of TMSOTf (each 100 μL of a stock solution containing 15 μL in 1 mL DCM, 0.032 mmol) were added during 5 h. The reaction was quenched by addition of triethylamine (22.6 μL), diluted with DCM and filtered over Celite®. The filtrate was concentrated and the residue was subjected to chromatography (hexane-EtOAc 3:1→1:2) to afford 69 mg of product containing minor amounts of the β-anomer. The crude was purified by HPLC (hexane-ether 2:3→1:2) to give pure compound **4** (48 mg, 58%) as syrup; $[\alpha]_D^{20}$ +11.4 (*c*, 2.1, $CHCl_3$). ^{1}H NMR (600 MHz, $CDCl_3$): δ 8.12 (dd, 2 H, *J* 8.3, 1.2 Hz, arom. H), 7.59 (br t, 1 H, *J* 7.5 Hz, arom. H), 7.47 (t, 2 H, *J* 7.7 Hz, arom. H), 5.67 (t, 1 H, $J_{4,3} = J_{4,5}$ 9.9 Hz, H-4), 5.37 (dd, 1 H, $J_{2,3}$ 3.2, $J_{2,1}$ 1.8 Hz, H-2), 5.24 (t, 1 H, $J_{4',3'} = J_{4',5'}$ 10.1 Hz, H-4'), 5.24-5.22 (m, 1 H, H-6'), 5.16 (dd, 1 H, $J_{2',3'}$ 2.9 Hz, H-3'), 5.00 (br m, 2 H, H-1', H-2'), 4.81 (d, 1 H, $J_{2,1}$ 1.7 Hz, H-1), 4.28 (dd, 1 H, $J_{7'a,6'}$ 7.2, $J_{7'a,7'b}$ 11.3 Hz, H-7'a), 4.27-4.19 (m, 4 H, H-6, H-5', H-3, H-7'b), 4.11 (dd, 1 H, $J_{7a,6}$ 8.7, $J_{7'a,7'b}$ 12.2 Hz, H-7a), 3.86 (dd, 1 H, $J_{7b,6}$ 1.2 Hz, H-7b), 3.75 (dd, 1 H, $J_{5,6}$ 1.7 Hz, H-5), 3.37 (s, 3 H, CH_3O), 2.14, 2.11, 2.09, 2.07, 1.98 and 1.91 (6 × s, each 3 H, 6 × CH_3CO), 1.12-0.93 [m, 28 H, 4 × C*H*(C*H*$_3$)$_2$]; ^{13}C NMR (125 MHz, $CDCl_3$): δ 170.37, 170.09, 169.83, 169.13, 169.07 and 166.11 (C=O), 133.49, 130.13, 12923 and 128.50 (Ar-C), 98.88 (C-1'), 98.50 (C-1), 75.00 (C-3), 73.76 (C-6), 71.53 (C-2), 71.28 (C-5), 70.08 (C-2'), 69.26 (C-5'), 68.24 (C-3'), 67.78 (C-7), 67.51 (C-6'), 67.32 (C-4), 65.10 (C-4'), 61.82 (C-7'), 55.15 (O*C*H$_3$), 20.89, 20.86, 20.65, 20.63 and 20.55 (*C*H$_3$CO), 17.63, 17.48, 17.46, 17, 39, 17.35, 17.32, 17.25 and 16.99 (TIPDS-*C*H$_3$), 13.15, 13.06, 12.67 and 12.56 (TIPDS-*C*H).

Methyl (2,3,4,6,7-penta-O-acetyl-L-glycero-α-d-manno-heptopyranosyl)-(1→3)-4-O-acetyl-2-O-benzoyl-L-glycero-α-D-manno-heptopyranoside (**C17**). A solution of **C16** (41.1 mg, 40.5 μmol) in dichloromethane (1.5 mL) was placed in a Teflon flask followed by addition of TREAT (0.15 mL, 0.92 mmol) at ice-bath temperature and the solution was then stirred for 20 h at rt. Ice-cold satd aq $NaHCO_3$ (20 mL) and EtOAc (20 mL) were added. The aqueous phase was reextracted with EtOAc and the combined organic phase was washed with brine, dried (Na_2SO_4) and concentrated. The residue was purified by chromatography on Isolute SPE Column Flash Si II column (hexane-EtOAc

1:2→EtOAc) to give **C14** (27.9 mg, 89%) as colorless syrup; $[\alpha]_D^{20}$ −0.5 (*c*, 1.0, $CHCl_3$). ^1H NMR (600 MHz, $CDCl_3$): δ 8.10 (br d, 2 H, *J* 7.4 Hz, Ar-H), 7.60 (dt, 1 H, *J* 7.4, 1.3 Hz, Ar-H), 7.49 (t, 2 H, *J* 7.9 Hz, Ar-H), 5.44 (dd, 1 H, $J_{2,3}$ 3.2, $J_{2,1}$ 1.7 Hz, H-2), 5.41 (t, 1 H, $J_{4,3}$ = $J_{4,5}$ 10.0 Hz, H-4), 5.29 (t, 1 H, $J_{4',3'}$ = $J_{4',5'}$ 10.1 Hz, H-4'), 5.26 (ddd, 1 H, $J_{6',5'}$ 1.9, $J_{6',7'a}$ 6.6, $J_{6',7'b}$ 6.3 Hz, H-6'), 5.14 (br, 1 H, H-1'), 5.13 (dd, 1 H, $J_{2',3'}$ 3.4 Hz, H-3'), 5.03 (dd, 1 H, $J_{2',1'}$ 1.6 Hz H-2'), 4.87 (d, 1 H $J_{1,2}$ 1.5 Hz, H-1), 4.35 (dd, 1 H, $J_{2,3}$ 3.4, $J_{3,4}$ 9.9 Hz, H-3), 4.315 (dd, 1 H, $J_{7'a,6'}$ 6.9, $J_{7'a,7'b}$ 11.3 Hz, H-7'a), 4.29 (dd, 1 H, H-5'), 4.24 (dd, 1 H, $J_{7'b,6'}$ 6.3 Hz, H-7'b), 3.94-3.91 (m, 1 H, H-7a), 3.70 (d, 1 H, H-5), 3.68-3.63 (m, 2 H, H-6, H-7b), 3.39 (s, 3 H, OCH_3), 3.25 (br d, 1 H, *J* 5.9 Hz, OH), 2.17, 2.15, 2.13, 2.10, 2.01 and 1.92 (5 × s, each 3 H, CH_3); ^{13}C NMR (125 MHz, $CDCl_3$): δ 171.90, 170.36, 170.05, 169.78, 169.22 and 166.04 (C=O), 133.63, 130.09, 129.05 and 128.64 (Ar-C), 98.94 (C-1'), 98.79 (C-1), 73.24 (C-3), 71.34 (C-2), 70.18 (C-5), 69.94 (C-2'), 69.28 (C-5'), 69.15 (C-4), 68.86 (C-6), 68.24 (C-3'), 67.20 (C-6'), 64.85 (C-4), 63.22 (C-7), 61.60 (C-7'), 55.31 (O*C*H_3), 21.03, 20.90, 20.68, 20.65, and 20.55 (*C*H_3CO).

Methyl *L-glycero-α-D-manno-heptopyranosyl-(1→3)-L-glycero-α-D-manno-heptopyranoside* (**C2**). A solution of **C17** (13.0 mg, 16.8 μmol) in dry MeOH (1.3 mL) was stirred with 1 M methanolic NaOMe (168 μL) for 6 h at rt. The reaction was stopped by addition of ion-exchange resin (Dowex, H^+-form), filtered and the filtrate was concentrated. In order to remove methyl benzoate, the residue was dissolved in D_2O followed by extraction with diethyl ether (3 × 1.5 mL). The aqueous phase was filtered through a plug of glass-wool, stripped with argon to remove traces of ether and lyophilized to give 6.3 mg (90%) of **C17** as colorless syrup, $[\alpha]_D^{20}$ +87.4 (*c* 0.6, D_2O). ^1H NMR (600 MHz, D_2O): δ 5.11 (d, 1 H, $J_{1',2'}$ 1.6 Hz, H-1'), 4.71 (H-1, hidden under solvent peak), 4.03 (dd, 1 H, $J_{3',2'}$ 3.4 Hz, H-2'), 4.02-4.00 (m, 2 H, H-6, H-6'), 3.99 (dd, 1 H, $J_{3,2}$ 3.4, $J_{1,2}$ 1.8 Hz, H-2), 3.93 (t, 1 H, $J_{3,4}$ = $J_{4,5}$ 9.8 Hz, H-4), 3.87 (dd, 1 H, H-3'), 3.84 (t, 1 H, $J_{3',4'}$ = $J_{4',5'}$ 9.8 Hz, H-4'), 3.80 (dd, 1 H, $J_{3,2}$ 3.4 Hz, H-3), 3.73-3.61 (m, 5 H, H-7a, H-7b, H-5', H-7a', H-7'b), 3.56 (dd, 1 H, $J_{6,5}$ 1.4 Hz, H-5), 3.35 (s, 3 H, OCH_3); ^{13}C NMR (125 MHz, D_2O): δ 103.24 (C-1'), 101.71 (C-1), 79.05 (C-3), 72.61 (C-5'), 72.02 (C-5), 71.32 (C-3'), 70.87 (C-2'), 70.51 (C-2), 69.92 and 69.49 (C-6, C-6'), 66.87 (C-4'), 66.41 (C-4), 63.66 and 63.63 (C-7, C-7'), 55.58 (O*C*H_3). ESI-HRMS: *m/z* calcd for $C_{15}H_{28}O_{13}$ $[M-H]^-$: 415.1457, found 415.1455.

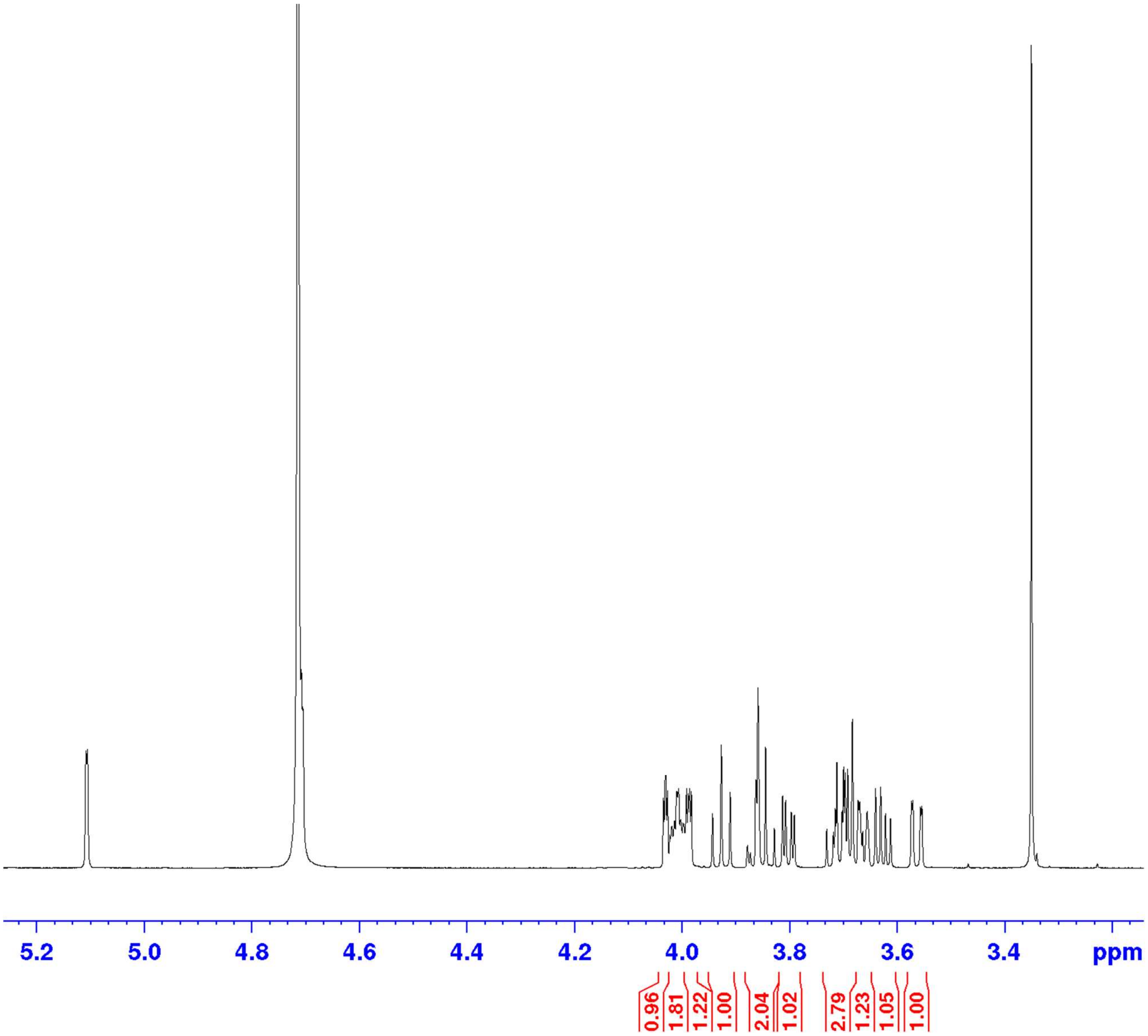

Figure S13. ^{1}H NMR spectrum of compound **C2** (600 MHz).

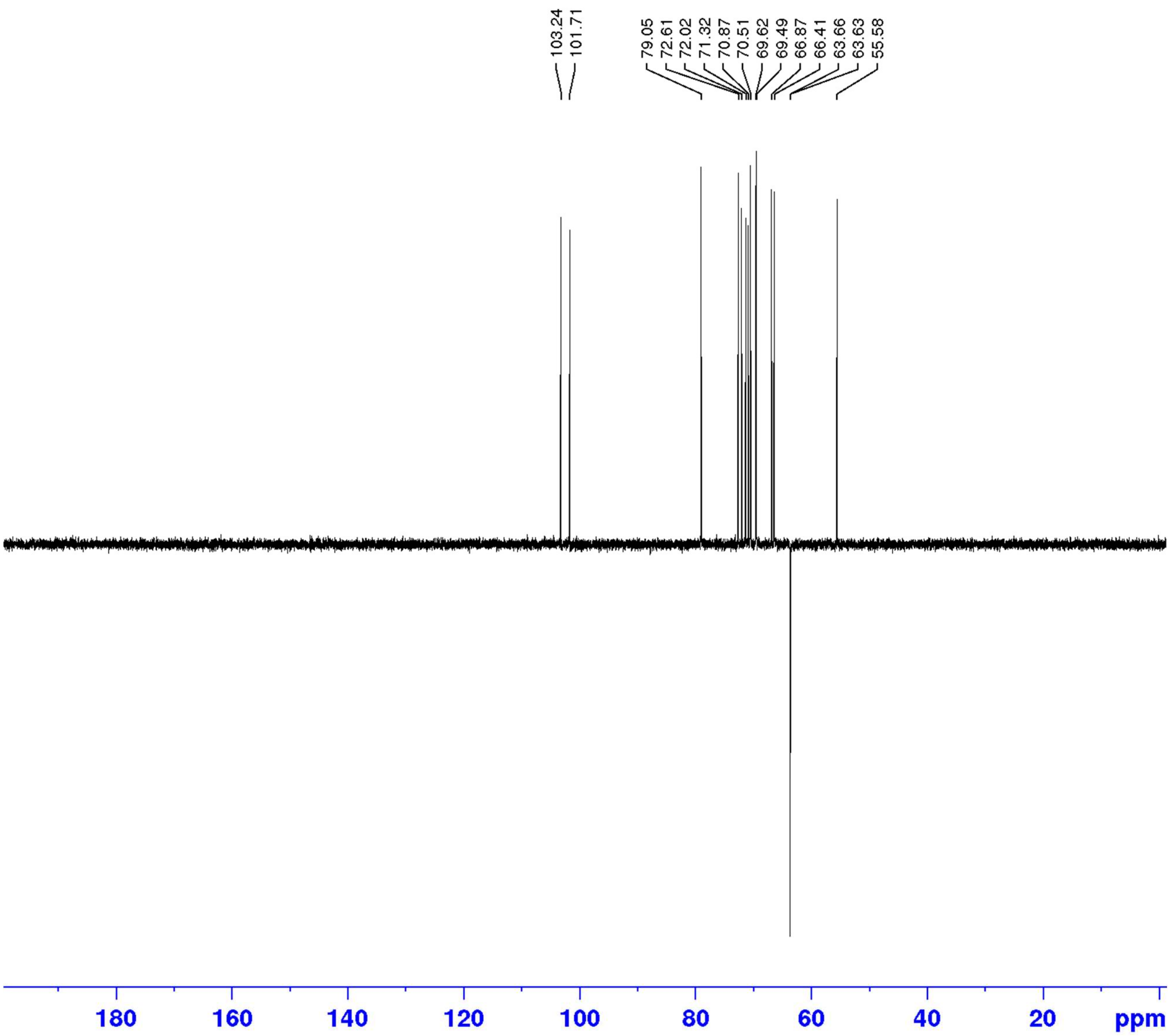

Figure S14. ^{13}C NMR spectrum of compound **C2** (APT, 125 MHz).

References

1. C. Stanetty, M. Walter, P. Kosma, Convergent Synthesis of 4-*O*-Phosphorylated L-*glycero*-D-*manno*-Heptosyl Lipopolysaccharide Core Oligosaccharides Based on Regioselective Cleavage of a 6,7-*O*-Tetraisopropyldisiloxane-1,3-diyl Protecting Group. *J. Org. Chem.* 2014 (*79*) 582-598. doi: 10.1021/jo402312x.

2. H. Paulsen, E. Höffgen, Synthesis of the heptose- and Kdo-containing trisaccharide inner-core region of lipopolysaccharides in immunogenic form. *Tetrahedron Lett.* 1991 (*32*) 2747-2750.
